# Feeding selenium-biofortified alfalfa hay during the preconditioning period improves growth, carcass weight, and nasal microbial diversity of beef calves

**DOI:** 10.1371/journal.pone.0242771

**Published:** 2020-12-01

**Authors:** Jean A. Hall, Anitha Isaiah, Gerd Bobe, Charles T. Estill, Janell K. Bishop-Stewart, T. Zane Davis, Jan S. Suchodolski, Gene J. Pirelli

**Affiliations:** 1 Department of Biomedical Sciences, College of Veterinary Medicine, Oregon State University, Corvallis, OR, United States of America; 2 Gastrointestinal Laboratory, College of Veterinary Medicine, Department of Small Animal Clinical Sciences, Texas A&M University, TX, United States of America; 3 Department of Animal and Rangeland Sciences, College of Agricultural Sciences, Oregon State University, Corvallis, OR, United States of America; 4 Linus Pauling Institute, Oregon State University, Corvallis, OR, United States of America; 5 Department of Clinical Sciences, College of Veterinary Medicine, Oregon State University, Corvallis, OR, United States of America; 6 USDA-ARS-Poisonous Plant Research Lab, Logan, UT, United States of America; Washington State University - Spokane, UNITED STATES

## Abstract

We previously reported that feeding Se-biofortified alfalfa hay to weaned beef calves in a preconditioning program decreases morbidity and mortality during the feedlot period. To understand the mode of action by which supranutritional Se supplementation supports calf health, we examined the effect of agronomic Se-biofortification on nasal microbiome and fecal parasites. Recently weaned Angus-cross beef calves (n = 30) were randomly assigned to two groups and fed an alfalfa hay-based diet for 9 weeks in a preconditioning program. Alfalfa hay was harvested from fields fertilized with sodium selenate at a rate of 0 or 90 g Se/ha. Calculated Se intake from dietary sources was 1.09 and 27.45 mg Se/calf per day for calves consuming alfalfa hay with Se concentrations of 0.06 and 3.47 mg Se/kg dry matter, respectively. Feeding Se-biofortified alfalfa hay for 9 weeks was effective at increasing whole-blood Se concentrations (556 ± 11 vs 140 ± 11 ng/mL; *P* < 0.001) and increasing body weight (*P*_*Treatment*,_ = 0.03) in weaned beef calves. Slaughter yield grades were higher for calves that had been fed Se-enriched alfalfa hay during the preconditioning period (*P*_*Treatment*_ = 0.008). No significant differences were observed in fecal parasite load, which remained low. The nasal microbiome and microbiota diversity within calves and across calves expanded from weaning (week 0) to the feedlot period (week 12), which was promoted by feeding Se-biofortified alfalfa hay. Especially concerning was the expansion of nasal Mycoplasmataceae in the feedlot, which reached over 50% of the total microbiota in some calves. In conclusion, we identified dietary Se-biofortified alfalfa hay as a potential promoter of nasal microbiome genome and microbiota diversity, which may explain in part high-Se benefits for prevention of bovine respiratory disease complex in beef calves.

## Introduction

Selenium (Se) is an essential trace mineral important for immune function and overall health of cattle. Optimal immune function is critical for calves undergoing the stresses of weaning, relocation to feedlots, and commingling with animals of different origins. The National Agriculture Statistics Service reports that 630,000 calves were born in Oregon in 2017. The majority of Oregon grown calves enter a feedlot. Even with good vaccination programs, calves often experience negative health issues in the feedlot, including mortality. Bovine respiratory disease (BRD) complex is a major cause of morbidity [1, 2]. Reducing morbidity and mortality losses by enhancing immune function would have a substantial economic impact for Oregon cattle producers.

Selenium has been recognized for years as an essential trace element for animals. The Northwest region is among those with the lowest amounts of Se in soils and plants [3]. In general, the majority of herbivorous livestock raised in low-Se regions do not receive sufficient dietary Se for optimum health. In Oregon, Se concentration of forages is lower than that required by livestock [4]. Providing adequate Se is important to prevent Se-responsive diseases. Although the essentiality of Se supplementation has been known for five decades, the most effective method of Se delivery to livestock to achieve optimum performance is still being investigated. Attempts to provide supplemental Se to animals through trace mineral supplementation or injection usually fail to maintain consistent blood Se concentrations necessary for optimal health and productivity. A promising Se supplementation method is Se fertilization, as it increases Se concentrations in plants, and consequently, in animals consuming the Se-biofortified forages [5, 6].

Plants require sulfur, not Se, for amino acid production. In plants, however, sulfur is replaced by Se resulting in selenomethionine. When Se-biofortified forage is consumed by livestock, Se from selenomethionine is incorporated into selenoproteins, whose functions range from antioxidant, anti-inflammatory, and detoxification to thyroid hormone activation. Nitrogenous fertilizers, widely hailed as one of the most important advances in agricultural technology, increase biomass but dilute essential minerals like Se, emphasizing the need for Se amendments [7]. Application of Se directly to pastures and hayfields increases forage Se concentration in a dose dependent manner [4] and improves blood Se concentrations [8], animal performance [6] and immunity [9, 10] in animals consuming that hay.

Both anthelmintic resistance and strict limits on the use of antibiotics emphasize the need for alternative methods for beef producers to enhance cattle immunity, prevent disease, and improve production. The objectives of this study were to show that feeding Se-biofortified hay increases whole-blood (WB) Se concentrations, decreases gastrointestinal parasite load, enriches the nasal microbial diversity, improves calf performance, and aids in disease prevention in the feedlot.

## Methods

### Animal ethics statement and study design

The experimental protocol was reviewed and this study was approved by the Oregon State University Animal Care and Use Committee (ACUP Number: 4883). This was a prospective clinical trial of 9-weeks duration (October 11, 2017 through December 11, 2017) involving 30 weaned beef calves (all steers), primarily of Angus breeding. The study design consisted of 2 treatment groups, with three pens of five animals per treatment. The study was conducted at the Hogg Animal Metabolism barn on the Oregon State University campus (Corvallis, OR, USA).

Corvallis is located at an elevation of 72 m within the Marine West Coast climate zone. Temperatures are mild year round, with warm, sunny summers and mild, wet winters with persistent overcast skies. Because of its close proximity to the coast range, temperatures dropping below freezing are uncommon. Average monthly temperatures for November are 10.8°C (high) and 3.3°C (low). Rainfall total is 110.9 cm/y. Typical distribution of precipitation includes about 50% of the annual total from December through February, lesser amounts in the spring and fall, and very little during summer.

The weaned beef calves at baseline ranged in age from 6 to 9 months and originated from the Oregon State University beef ranch, Corvallis, OR, USA. Body weights at baseline ranged from 264 to 369 kg (328 ± 5.0 kg, mean ± SEM), and body condition scores ranged from 6 to 7 (1 to 9 scale). Routine farm management practices, including vaccinations and deworming, were the same for both treatment groups.

Using a randomized complete block design, calves were blocked at the time of weaning by body weight (BW) and then assigned to one of 2 treatment groups of 15 calves each. Ear tags were used to identify calves. Calves were then placed by treatment group into pens (3 pens of 5 calves/treatment group). Pens provided 10 m^2^/calf of concrete flooring in open lots that were strip cleaned once weekly, 5 m^2^/calf bedded with wood shavings in a loafing area, and 98 cm of feeder space/calf as concrete bunks. All measurements exceeded requirements [11] with continuous access to water, feed bunks, and shelter.

Calves were fed a mixture of alfalfa and grass hay twice daily. The amount of hay fed was adjusted weekly to ensure that calves had all the hay they wanted for consumption with minimal wastage. The ration was formulated for growing beef calves in the 250 to 350 kg BW range to achieve a target average daily gain of 0.5 kg/day. The goal was to feed hay composed of 85% alfalfa and 15% grass hay. Calves were transitioned to their respective hay rations over a 3-week period. Alfalfa hay was fed as follows: 0.68 kg/calf on day 1; 1.14 kg/calf on day 2; 1.59 kg/calf on day 3; 2.05 kg/calf on day 4; 2.5 kg/calf on day 5; 2.95 kg/calf on day 6; and 3.41 kg/calf on day 7. During the first week, grass hay was added to achieve a total hay intake of 6.82 kg/calf per day. Thereafter, in week 2 the amount of alfalfa hay fed was increased from 3.41 to 5.91 kg/calf per day. In week 3 alfalfa hay fed was increased to 6.36 kg/calf per day and total hay intake averaged 7.73 kg/calf per day. In weeks 4, 5, and 6 alfalfa hay fed was increased from 6.36 to 7.27 kg/calf per day and total hay intake was increased from 7.73 to 8.64 kg/calf per day. In weeks 7, 8, and 9 alfalfa hay intake was 7.73 kg/calf per day and total hay intake was between 8.18 and 9.09 kg/calf per day.

Also in week three, 0.45 kg/calf per day of a medicated grain-based concentrate was fed (OSU Steer-A-Year Pellet R35; manufactured by CHS Inc., Sioux Falls, SD). This amount was increased to 0.68 kg/calf per day in weeks 4 through 9. This feed contained a coccidiostat (monensin sodium, 35 g/ton). The grain pellets were fed once a day and consumed before hay was fed. The guaranteed analysis of grain pellets was 11.0% crude protein, 3.5% crude fat, and 6.0% crude fiber. The concentration of Se in the grain pellets was 0.77 mg/kg.

Prior to this study, calves had free-choice access to a mineral supplement containing 120 mg/kg Se from sodium-selenite. The mineral supplement (dry matter, DM basis) was in loose granular format and contained 57.0 to 64.0 g/kg calcium; 30.0 g/kg phosphorus; 503 to 553 g/kg salt (NaCl); 50.0 g/kg magnesium; 50 mg/kg cobalt; 2,500 mg/kg copper; 200 mg/kg manganese; 200 mg/kg iodine; 6,500 mg/kg zinc (Wilbur-Ellis Company, Clackamas, OR). During the feeding trial, this same mineral supplement without Se was provided for free-choice consumption.

Health was monitored daily during the 9-week preconditioning period. We looked for adverse health events such as being off feed, fever, respiratory distress, diarrhea, abscess, pink eye, and lameness. After the 9-week preconditioning period, calves were shipped to a commercial feedlot in Burbank, WA (Simplot Feeders, Pasco WA). Calves were physically examined and nasal swabs and blood and fecal samples were collected 3 weeks after transfer to the feedlot. Health information was available through 5 weeks after transfer to the feedlot. At the end of the 25-week feedlot period, calves were sent to a commercial meat packing plant (Tyson Fresh Meats Inc.; Pasco, WA). Hot carcass weights, carcass quality grades (no-roll, standard, select, choice, prime), and yield grades (1 to 4) were recorded.

### Selenium biofortified-alfalfa hay preparation and analyses

Third cutting alfalfa hay was enriched with Se by mixing inorganic sodium-selenate (RETORTE Ulrich Scharrer GmbH, Röthenbach, Germany) with water and spraying it onto the soil and foliage (approximately 10 cm height) of an alfalfa field at application rates of 0 or 90 g Se/ha in July 2017. The application rate was chosen based on a previous study [6]. Third-cutting alfalfa hay was harvested early October 2017 and then analyzed for Se and nutrient content. A Penn State forage sampler was used to take 25 cores from random bales in each hay source (0 or 90 g Se/ha) prior to beginning the feeding trial. Core samples were mixed well and representative samples selected for Se analysis (Table 1; Utah Veterinary Diagnostic Laboratory, Logan, UT). Plant samples were prepared for Se analysis as previously described [12], and Se was determined using inductively coupled argon plasma emission spectroscopy (ICP-MS; ELAN 6000, Perkin Elmer, Shelton, CT). Quantification of Se was performed by the standard addition method, using a 4-point standard curve. A quality-control sample (in similar matrix) was analyzed after every 5 samples, and Se analysis was considered acceptable if the Se concentration of the quality-control sample fell within ± 5% of the standard/reference value for the quality control. Alfalfa hay samples were also submitted to a commercial laboratory for routine nutrient analysis (Table 1; Cumberland Analytical Services, Waynesboro, PA). Alfalfa hay dry-matter determination was completed at a temperature of 105°C for 12 to 14 h in a forced draught oven. Methods for crude protein (CP), acid detergent fiber (ADF), ash, and minerals were performed according to the Association of Official Analytical Chemists [13]. The neutral detergent fiber (NDF) was determined according to Van Soest et al. [14]. Soluble protein was determined according to Krishnamoorthy et al. [15].

**Table 1:** Alfalfa and grass hay nutrient compositions (DM basis)^1^^,^^2^^,^^3^.

Nutrient	Alfalfa Hay		Grass Hay
	Control	High-Se	
Dry matter, g/kg	842	870	883
Crude protein, g/kg	163	170	75
Acid detergent fiber, g/kg	317	332	435
Neutral detergent fiber, g/kg	390	393	668
Nonfiber carbohydrates, g/kg	371	343	195
Fat, g/kg	0.2	< 0.1	0.1
Ash, g/kg	75.8	95.1	61.9
TDN, g/kg	631	609	549
Calcium, g/kg	17.2	18.0	4.8
Phosphorus, g/kg	2.4	2.4	1.6
Magnesium, g/kg	5.3	5.4	2.6
Potassium, g/kg	9.7	10.0	6.0
Sodium, g/kg	3.9	3.8	3.2
Copper, mg/kg	14	15	6
Iron, mg/kg	627	1196	214
Manganese, mg/kg	39	56	116
Zinc, mg/kg	19	21	28
Selenium, mg/kg	0.06	3.47	0.12

^1^Alfalfa and grass hay samples were submitted to Cumberland Valley Analytical Services, Waynesboro, PA for routine nutrient analysis, and to Utah Veterinary Diagnostic Laboratory, Logan, UT for Se analysis.

^2^Alfalfa and grass hay DM determination was completed at a temperature of 105°C for 12 to 14 h in a forced draught oven. Methods for CP, ADF, ash, and minerals were performed according to the Association of Official Analytical Chemists [13]. The NDF was determined according to Van Soest et al. [14]. Soluble protein was determined according to Krishnamoorthy et al. [15].

^3^Alfalfa and grass hay samples were prepared for Se analysis as described by Davis et al. [12], and Se determined using inductively coupled argon plasma emission spectroscopy (ICP-MS; ELAN 6000, Perkin Elmer, Shelton, CT).

### Blood Se collection and analyses

Blood samples were collected from the jugular vein of weaned beef calves, at baseline, after 3, 6, and 9 weeks of alfalfa hay consumption, and 3 weeks after transfer to the feedlot, into evacuated EDTA tubes (2 mL; final EDTA concentration 2 g/L; Becton Dickinson, Franklin Lakes, NJ) and stored on ice until they were frozen at -20°C to measure WB-Se concentrations. Selenium concentrations were determined by a commercial laboratory (Utah Veterinary Diagnostic Laboratory, Logan, UT) using an ICP-MS (ELAN 6000, Perkin Elmer, Shelton, CT) method as previously described [6].

### Fecal collection and analyses

Fecal samples were collected 30 days before the feeding trial began, after 5 and 9 weeks of alfalfa hay consumption, and again 3 weeks after transfer of calves to the feedlot. Fecal samples were extracted manually per rectum using a powder-free latex examination glove (Diamond Grip^TM^ Latex Gloves, Microflex, Reno, NV) while calves were restrained in a chute. Fecal samples were stored in the glove at 4°C until analysis. The minimum sample amount was 10 grams.

For quantification of fecal trichostrongyle-type egg counts, we followed the McMaster egg count method of Whitlock [16]. In brief, 2 grams of feces were mixed with a mortar and pestle in 13 mL of saturated NaCl solution in order to facilitate the release of the ova from the fecal material and to prepare a homogeneous solution of the feces. The suspension was poured through food grade cheese cloth to remove large particles. Using a disposable Pasteur pipette (GeneMate, BioExpress, Kaysville, UT), we transferred an aliquot of the fecal solution to the chambers of a McMaster slide (Chalex Corp., Wallowa, OR). The slide was examined under low power (100X) by focusing on the etched lines on the underside of the top chamber. Both chambers on the McMaster’s slide were counted and the number of trichostrongyle-type eggs was multiplied by 25 to obtain the number of eggs/g of feces. Counting was complete within 2 days of sample collection.

When at least 25 trichostrongyle-type eggs/g feces were observed, fecal samples were submitted to a commercial laboratory (Veterinary Diagnostics Laboratory, Oregon State University) and assayed for the presence of *H*. *contortus* eggs using a fluorescein-labeled peanut agglutinin test, following the procedure of Jurasek et al. [17]. In brief, 2 grams of fecal matter were placed in 5 mL of water and crushed to break up the fecal material. Next, 93 mL of water was added (for a total of 98 mL water) and mixed with the fecal material. The sample was then refrigerated overnight to allow the eggs to separate from the fecal matter. The next morning, the sample was thoroughly mixed to form a uniform suspension and immediately decanted into a centrifuge tube, which was spun at 280 × g for 5 minutes. The supernatant was decanted and 2 mL of Stoll’s saturated sugar solution (specific gravity = 1.27) [18] was added and mixed with the fecal pellet. The tube was then filled to within a few mm from the top with the sugar solution and centrifuged at 280 × g for 5 minutes. The tube was placed in a stable upright rack and filled with additional sugar solution until a convex meniscus formed, and then a 22 mm^2^ coverslip was placed on top. The solution sat for 1 hour to allow eggs to float to the top of the tube and collect on the coverslip. The coverslip was then gently removed and rinsed with 100 mM 7.4 pH phosphate buffered saline (PBS) into a 1.5 mL Eppendorf tube. The PBS solution was added to 1.5 mL, and the sample was rinsed by centrifuging at 280 × g for 5 minutes. The supernatant was removed and the egg sediment resuspended in an additional 1.5 ml of PBS. Eggs in PBS were centrifuged again at 280 × g for 5 minutes, the supernatant removed, and the pellet resuspended in 1mL of fluorescein isothiocyanate-labeled peanut agglutinin (Sigma Cat. No. L-7381 lectin from *Arachis hypogaea*, reconstituted at 5 μg/1 mL in PBS; Sigma, St. Louis, MO). Eggs then were incubated in the lectin for 1 hour under constant agitation at room temperature. Samples were washed twice more in PBS, as described above, and 5 μL of the egg sediment was transferred onto a glass slide with 3 μL of fluorescent mounting fluid (Veterinary Medical Research and Development Inc., Pullman, WA) and then overlaid with a coverslip. Specimens were examined using a fluorescence microscope with fluorescein isothiocyanate filters (480–490 excitation/527/30 emission). The percentage of total ova that were positively identified as *H*. *contortus* was reported. If large numbers of ova were present, this was based on counting 100 eggs.

### Nasal microbiota collection and analyses

Nasal swabs were collected from all calves 30 days before the feeding trial began, after 9 weeks of alfalfa hay consumption, and again 3 weeks after transfer to the feedlot. Sterile, individually wrapped, polyester tipped applicators (Puritan®, Guilford, ME) were inserted approximately 10 cm into the ventral meatus of the nares, twirled to collect a mucosal swab, and then placed into individual sterile containers (10 mL, red top, BD Vacutainer collection tubes; Becton Dickinson, Franklin Lakes, NJ) avoiding any contamination or contact with the plastic stick. Swabs in tubes were subsequently frozen at -80^ᵒ^C within 4 hours of collection. Negative control (sterile) swabs were similarly processed.

Microbial DNA was extracted from nasal swab samples using MoBio Power soil DNA isolation kit (MoBio Laboratories, USA) as per the manufacturer’s instructions. The V4 region of the 16S rRNA gene was amplified with primers 515F (5′-GTGCCAGCMGCCGCGGTAA-3′) and 806R (5′-GGACTACVSGGGTATCTAAT-3′) at the MR DNA Laboratory (Shallowater, TX, USA) and sequenced on an Illumina MiSeq instrument at the MR DNA Laboratory (Shallowater, TX, USA).

The sequencing data were processed using the QIIME 2 v 2018.6 (https://qiime2.org/) platform [19]. The raw reads were deposited in NCBI SRA under the accession number SRP144214. Raw sequence data were de-multiplexed with the q2-demux plugin in QIIME2. The data was quality filtered by removing low quality regions and chimeric sequences using the Divisive Amplicon Denoising Algorithm 2 (DADA2) with the q2-dada2 plugin [20] to create an amplicon sequence variant (ASV) table. A masked alignment of the sequence variants was conducted using MAFFT [21] with the q2-alignment plugin. A phylogeny tree was created with FastTree2 [22] using the q2-phylogeny plugin. Furthermore, taxonomy was assigned using the QIIME2 naive Bayes feature classifier [23] trained on the Greengenes 13_8 database [24]. The feature table was also filtered to remove sequences that were classified as mitochondria and chloroplasts.

Sequences were rarefied to an even depth of 31,744 sequences per sample to account for unequal sequencing depth across samples in QIIME 2 using the “feature-table rarefy” plugin. The q2-diversity plugin was used to calculate measures of alpha-diversity (observed ASVs, Chao1, Shannon index) and beta diversity (weighted and unweighted UniFrac distances, Bray-Curtis Dissimilarities).

To determine if there were any changes in microbial function in the nasal microbiota, PICRUSt2 [25] (Phylogenetic Investigation of Communities by Reconstruction of Unobserved States) was applied to make functional gene content predictions on the 16S rRNA gene sequencing data. The standard pipeline was implemented. The functional profiles were obtained with PICRUSt2 using standard pipeline and using the default options with picrust2_pipeline.py.

### Statistical analyses

Data for Se status and growth characteristics were analyzed as repeated-measures-in-time using PROC MIXED in SAS version 9.2 [26]. Data were averaged within feeding pens. Fixed effects in the model were Se application rate (0 and 90 g Se/ha), baseline values (as linear covariate), time (after 3, 6, and 9 weeks of feeding Se enriched hay), and the interaction between Se application rate and time. An unstructured variance-covariance matrix was used to account for variation of measures within pens. The Kenward-Rogers option was used to account the degrees of freedom for repeated measures within time. Carcass and other data collected after the preconditioning period were analyzed in PROC GLM with treatment as fixed effect.

Fecal parasite egg counts were logarithmically transformed by base 10 to achieve normal distribution. Data were measured over time; thus, repeated-measures-in-time analysis was performed using PROC GLIMMIX in SAS, assuming a negative binomial distribution. Fixed effects in the model were Se application rate (0 and 90 g Se/ha), baseline values (as linear covariate), time (after 5 and 9 weeks of feeding Se enriched hay), and the interaction between Se application rate and time.

Data analysis of nasal swabs was performed on the rarefied, total sum scaling (TSS) normalized ASV table using Calypso v 8.84 [27]. The diversity of bacterial communities within calves (alpha diversity) were determined using the Chao1 index and the observed ASVs, which both measure the presence of bacterial taxa, and the Shannon index, which also accounts for the relative abundance of bacterial taxa [28, 29]. Differences in bacterial communities between calves and within calves across time (beta diversity) were determined using the Bray Curtis dissimilarity distance metric for functional gene data and the phylogeny-based unweighted and weighted UniFrac distance metrices for other microbiome and microbiota data [30–32]. Unweighted UniFrac distances measure differences in the presence of bacterial taxa, whereas weighted UniFrac distances measure not only differences in the presence of bacterial taxa but also in the relative abundance of bacterial taxa. The diversity measures and the number of bacterial taxa and functional genes present were analyzed as repeated-measures-in-time using PROC MIXED in SAS. Fixed effects in the model were Se application rate (0 and 90 g Se/ha), time (weeks 0, 9, and 12), and the interaction between Se application rate and time. An unstructured variance-covariance matrix was used to account for variation of measures within calves. The Kenward-Rogers option was used to account the degrees of freedom for repeated measures within time.

Principal coordinate analysis (PCoA) plots for unweighted UniFrac distances and Bray Curtis dissimilarities were generated with QIIME2. The ANOSIM (Analysis of Similarity) test within PRIMER 6 software package (PRIMER-E Ltd., Luton, UK) was performed on the weighted and unweighted UniFrac and Bray Curtis distance metrics to find significant differences in microbial communities between groups. To compare bacterial taxa between groups and across time, the non-parametric Kruskal Wallis rank sum test was used for bacterial taxa that were present in 14 or more of 15 calves of Control and High-Se groups in SAS. The non-parametric Fisher’s Exact test was used for bacterial taxa that were present in less than 14 calves of Control and High-Se groups in SAS. In addition, Linear discriminant analysis effect size (LEfSe) was utilized within Calypso [27] to identify bacterial taxa that were differentially abundant between Control calves and calves fed High-Se enriched alfalfa hay, with an LDA score threshold of > 3.0 (results not shown). All statistical tests were two-sided. Data are reported as least square mean ± SEM. Statistical significance was declared at *P* ≤ 0.05 and a tendency at 0.05 < *P* ≤ 0.10.

## Results

### Effect of feeding weaned beef calves Se-biofortified alfalfa hay in a preconditioning program on Se intake

Fertilizing the alfalfa hay field with sodium-selenate at 90 g/ha increased the Se content of third-cutting alfalfa from 0.06 (non-fertilized control) to 3.47 mg Se/kg DM. Calculated Se intake from alfalfa hay was 0.46 and 26.82 mg Se/calf per day, respectively, for calves consuming hay with Se concentrations of 0.06 and 3.47 mg Se/kg DM. The concentration of Se in the grass hay was 0.12 mg/kg. Calculated Se intake from grass hay was approximately 0.11 mg Se/calf per day. The measured Se concentration of the grain concentrate was 0.77 mg/kg DM. Calculated Se intake from grain concentrate was 0.52 mg Se/calf per day in weeks 7 through 9 of the preconditioning period. Thus, total dietary Se intake during weeks 7 to 9 was 1.09 and 27.45 mg Se/calf per day, respectively for calves in control and high-Se treatment groups.

### Effect of feeding weaned beef calves Se-biofortified alfalfa in a preconditioning program on Se status

**Fig 1:**
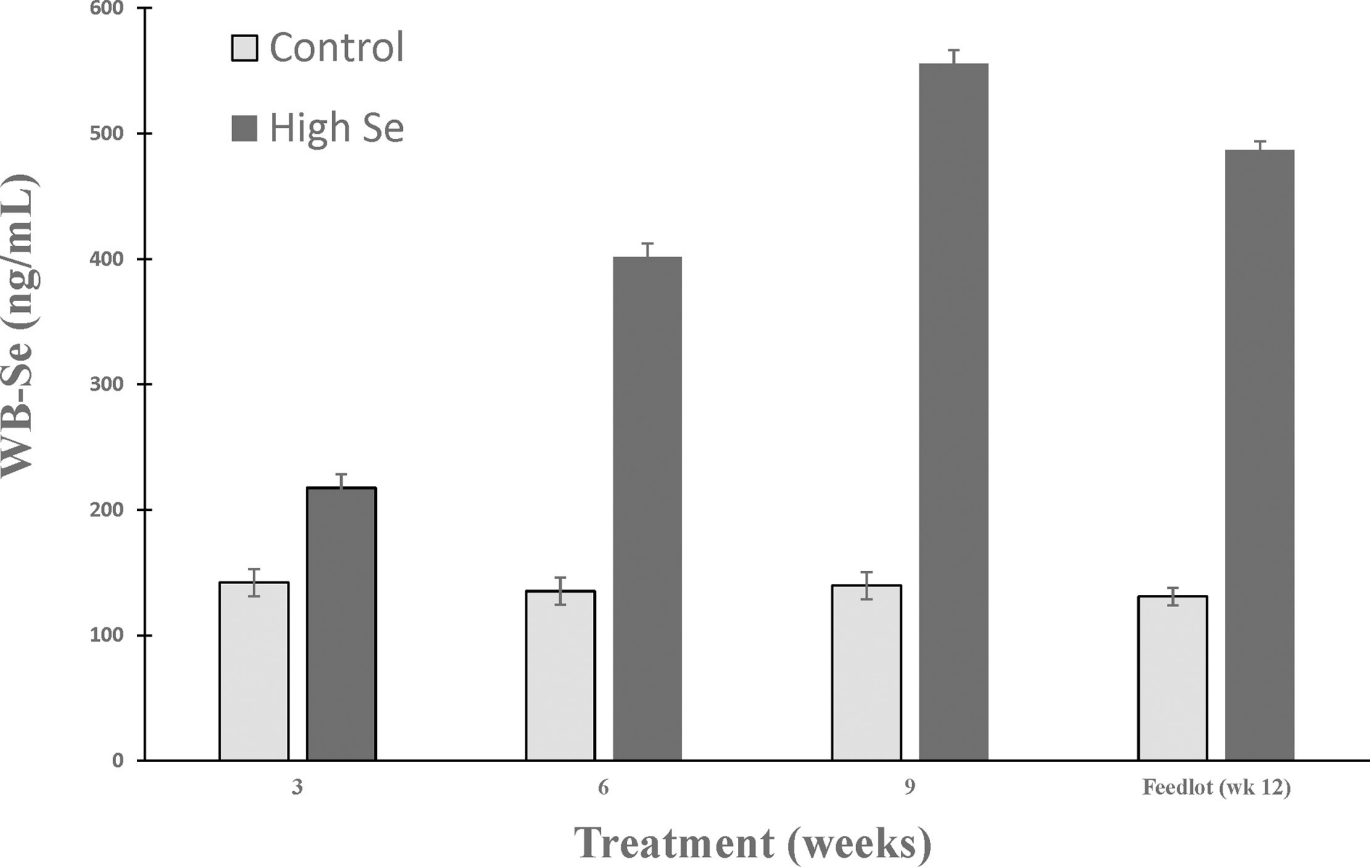
Comparison of whole-blood Se concentrations (mean ± SEM) in weaned beef calves after 3, 6, and 9 weeks in the preconditioning period, and in the feedlot (week 12). During the 9 week preconditioning period, calves consumed alfalfa hay harvested from a field not fertilized with Se (Control) or from a field fertilized with sodium-selenate (High Se; application rate of 90 g Se/ha; n = 15 calves per group for Control and High Se). Total dietary Se intake during weeks 7 to 9 was 1.09 and 27.45 mg Se/calf per day, respectively for calves in control and High-Se treatment groups. The normal reference interval for whole-blood Se concentrations in beef cattle is 120 to 300 ng/mL.

Feeding Se-fertilized alfalfa hay was effective at increasing WB-Se concentrations in weaned beef calves (*P*_*Treatment*,_ *P*_*Week*_, and *P*_*Interaction*_ all ≤ 0.004; Fig 1). The normal reference interval for WB-Se concentrations of adult cows is 120 to 300 ng/mL [5]. The WB-Se concentrations continued to increase throughout the 9-week preconditioning period (week 3: +50%; week 6: +192%; week 9: +292%). During the initial feedlot period (at week 12: +272%), WB-Se concentrations remained higher (*P*_*Treatment*_ < 0.001).

### Effect of feeding weaned beef calves Se-biofortified alfalfa hay in a preconditioning program on growth characteristics and carcass data

All calves remained healthy during the 9-week preconditioning period and were healthy upon physical examination 3 weeks after transfer to the feedlot. One calf was treated for a fever and hock injury 4 weeks after transfer to the feedlot. Otherwise, no calves experienced clinical signs of BRD through week 5 after transfer to the feedlot.

**Fig 2:**
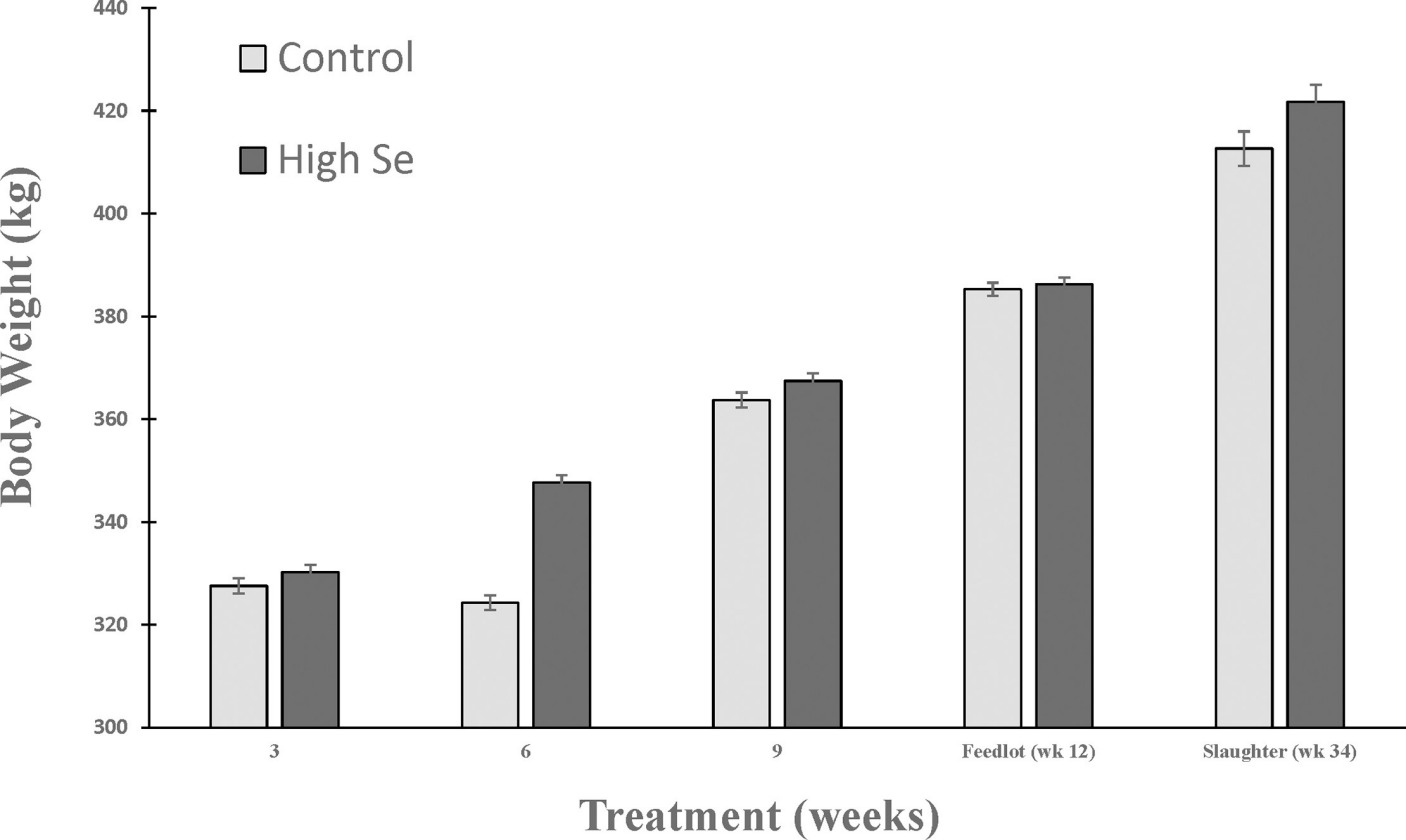
Comparison of baseline-adjusted BW (kg; mean ± SEM) of weaned beef calves (primarily of Angus breeding and ranging in age from 6 to 9 months at baseline). During the 9 week preconditioning period, calves consumed alfalfa hay harvested from a field not fertilized with Se (Control) or from a field fertilized with sodium-selenate (High Se; application rate of 90 g Se/ha; n = 15 calves per group for Control and High Se). Body weights at baseline ranged from 264 to 369 kg (328 ± 5 kg, mean ± SEM), and final body weights at the end of the preconditioning period ranged from 288 to 411 kg (366 ± 5 kg, mean ± SEM).

Feeding Se-fertilized alfalfa hay was effective at increasing BW in weaned beef calves (*P*_*Treatment*,_ = 0.03, *P*_*Week*_, < 0.001, and *P*_*Interaction*_ = 0.42) and tended to be effective at increasing hot carcass weight at week 34 (*P*_*Treatment*_ = 0.07; Fig 2). During the initial feedlot period (at week 12) no significant effect of feeding Se-enriched hay on BW was observed (+2.2%; *P*_*Treatment*_ = 0.58). At slaughter, no significant differences were observed for carcass quality grade levels [prime (highest), choice, select, standard, no-roll (lowest)], as both groups had all but 4 animals with choice grade (*P*_*Treatment*_ = 0.53). However, yield grade [1 (most desirable trim), 2, 3 (industry average), and 4] were improved in animals that were fed Se-enriched alfalfa hay during the preconditioning period (*P*_*Treatment*_ = 0.008), as more calves in the group fed Se-enriched alfalfa hay had a 1 or 2 (86%) compared with calves in the control group (36%). Slaughter data were not available for 4 calves in the control group and 1 calf in the high-Se treatment group because ear tags were lost at slaughter.

### Effect of feeding weaned beef calves Se-biofortified alfalfa hay in a preconditioning program on fecal parasite counts

We evaluated fecal parasite load at weeks 0, 5, and 9 (end) of a preconditioning period as well as in the feedlot (week 12). Overall, fecal parasite counts were low; especially counts for *Nematodirus*, *Maniezia*, and *Trichuris* were mostly zero. The range for coccidia oocyst counts were between 0 and 600 oocysts/g and for trichostrongyle-type egg counts were between 0 and 450 eggs/g. Feeding Se-fertilized alfalfa did not significantly alter coccidia (*P*_*Treatment*_ = 0.96) and trichostrongyle-type egg counts (*P*_*Treatment*_ = 0.81). No significant time, nor treatment × time interactions were observed. At the beginning of the feedlot period, calves were dewormed. As a result, fecal counts were zero or ≤ 25 eggs/g feces in week 12.

### Effect of feeding weaned beef calves Se-biofortified alfalfa hay in a preconditioning program on nasal microbiome genome

**Fig 3:**
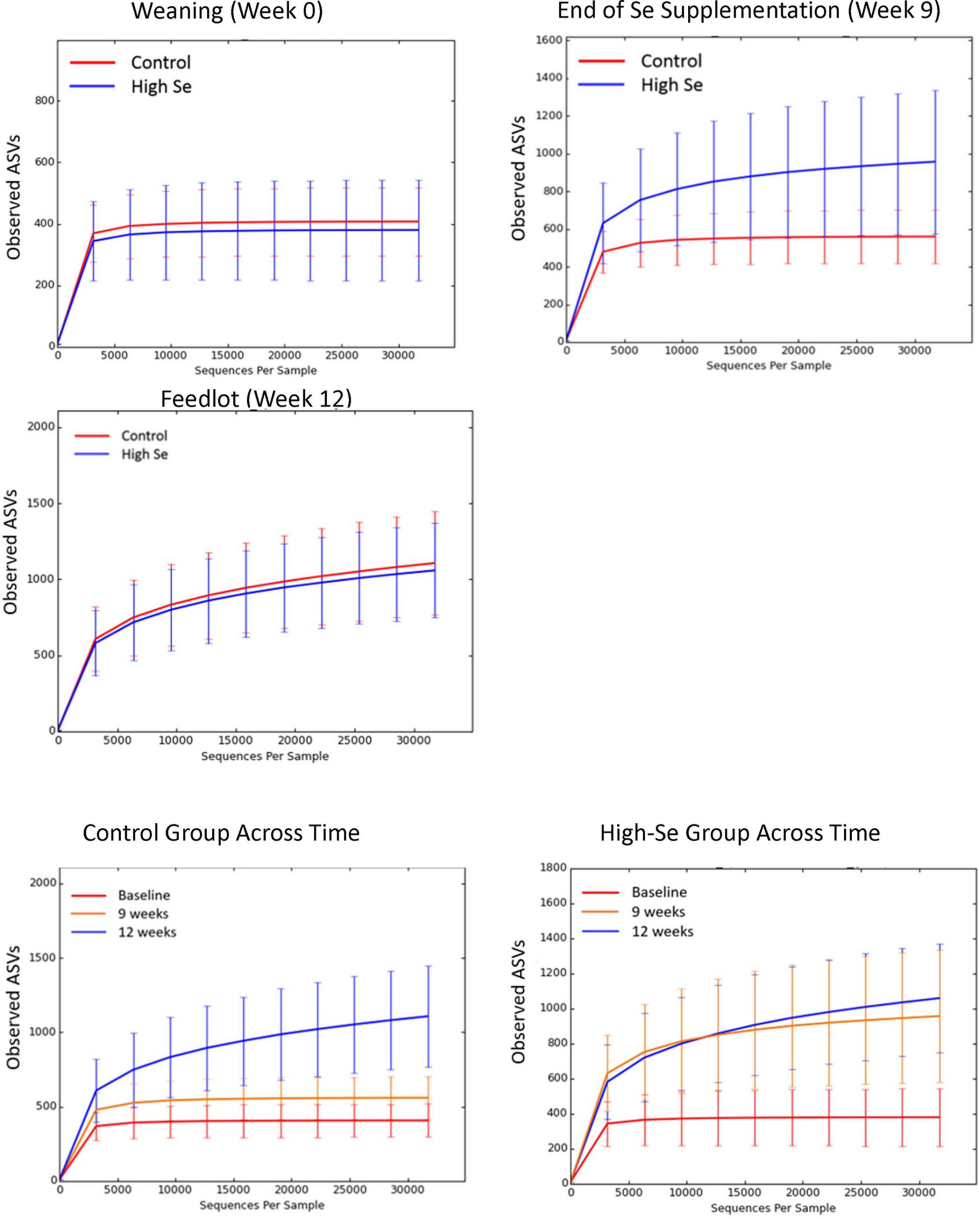
Rarefaction analysis of 16S rRNA amplicon sequence variants (ASVs) obtained from nasal swabs after weaning (week 0), at the end of the Se-supplementation preconditioning period (week 9), and in the feedlot (week 12). During the 9 week preconditioning period, calves consumed alfalfa hay harvested from a field not fertilized with Se (Control) or harvested from a field fertilized with sodium-selenate (High-Se; application rate of 90 g Se/ha; n = 15 calves per group for Control and High-Se).

We collected nasal microbiome genome at weeks 0 and 9 (end of the Se-supplementation preconditioning period) as well as in the feedlot (week 12). Sequencing of the bacterial 16S rRNA gene in the samples yielded 10,308,260 quality sequences (n = 90; mean ± SD, 107,377 ± 48,791). Rarefaction analysis was performed at a depth of 31,744 sequences (Fig 3). Expansion of the nasal microbiome diversity was promoted by feeding Se-biofortified alfalfa hay in the Se-supplementation preconditioning period as counts of 16S rRNA genes expanded within High-Se calves during the Se-supplementation preconditioning period (Fig 3A). Nasal microbiome diversity increased within Control calves during the transition to the feedlot (Fig 3A).

The microbial diversity increased within High-Se calves during the Se-treatment period, as indicated by Chao1 (+171%) and observed ASVs (+151%). Control calves caught up with High-Se calves in microbial diversity in the feedlot (Fig 3; Table 2). There were no significant shifts in the microbial diversity toward a more even microbial profile across treatment and time, as indicated by a non-significant Shannon index (Table 2).

**Table 2:** Effect of feeding Se-biofortified hay in a 9-week preconditioning period on nasal microbiome diversity within calves.

Alpha-diversity Measures*	Control (n = 15)	High Se (n = 15)	SEM	*P* value
**Chao1**				
Week 0 (Weaning)	408^b^	381^b^	38	0.60
Week 9 (End of Se Treatment)	561^b^	1034^a^	84	0.0005
Week 12 (Feedlot)	1367^a^	1288^a^	110	0.61
**Observed ASV**				
Week 0 (Weaning)	408^b^	381^b^	37	0.61
Week 9 (End of Se Treatment)	560^b^	956^a^	76	0.001
Week 12 (Feedlot)	1107^a^	1059^a^	87	0.70
**Shannon Index**				
Week 0 (Weaning)	7.90	7.75	0.17	0.55
Week 9 (End of Se Treatment)	8.14	8.40	0.27	0.50
Week 12 (Feedlot)	8.10	7.87	0.35	0.65

*Summary of alpha diversity measures at a rarefaction depth of 31,744 sequences per sample for Control and High-Se calves. Results are presented as least-squared means and pooled standard errors of the mean. LSMeans with different superscripts (a,b) differ across treatment and time at *P* < 0.01.

**Fig 4:**
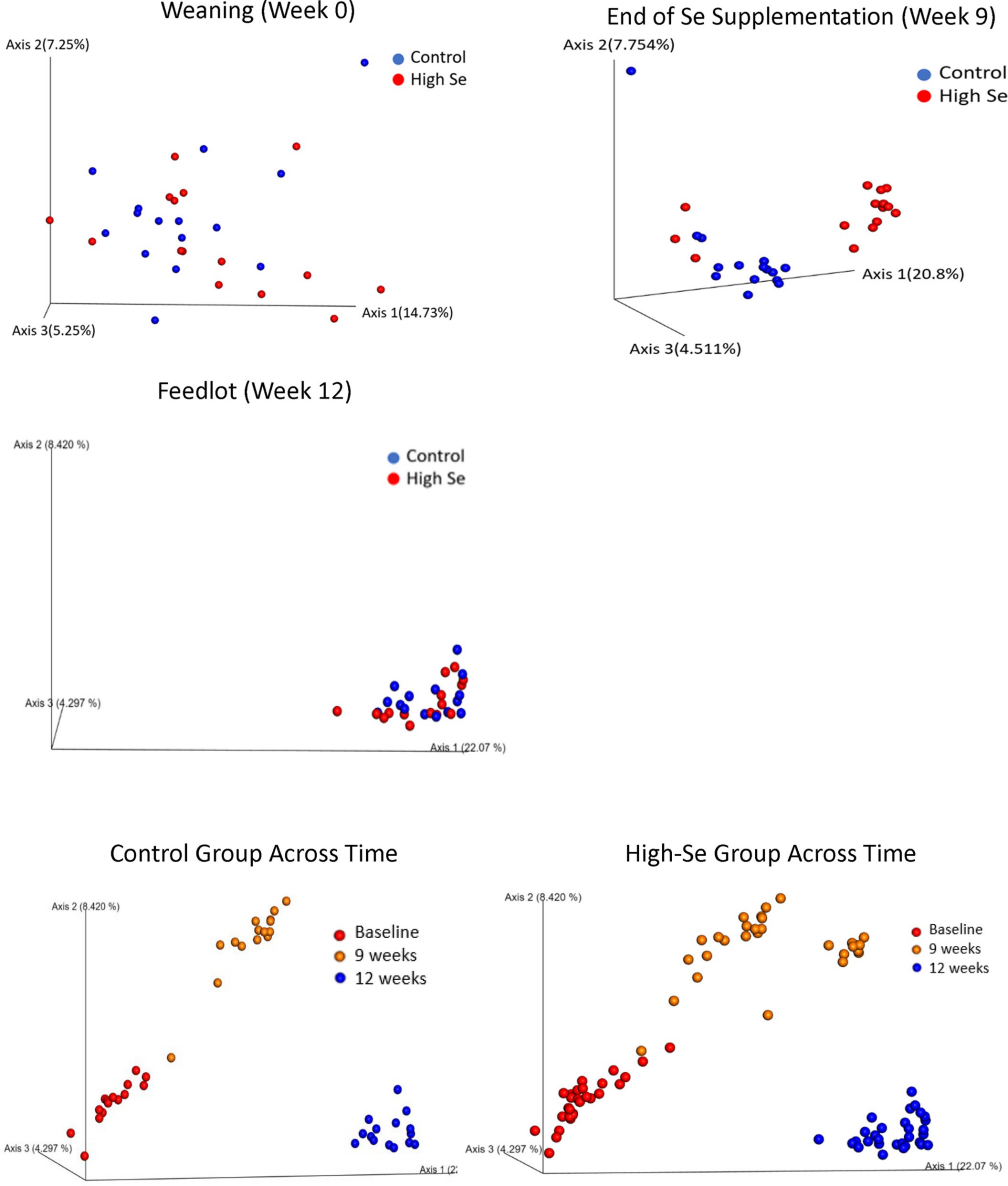
Principal coordinate analysis of unweighted UniFrac distances obtained from nasal swabs after weaning (week 0), at the end of the Se-supplementation preconditioning period (week 9), and in the feedlot (week 12). During the 9 week preconditioning period, calves consumed alfalfa hay harvested from a field not fertilized with Se (Control) or harvested from a field fertilized with sodium-selenate (High Se; application rate of 90 g Se/ha; n = 15 calves per group for Control and High Se).

Nasal microbiome differed between High-Se and Control calves at the end of the Se-treatment preconditioning period (week 9), as shown by the distinct clustering of High-Se and Control calves within the principal coordinates (Fig 4) and ANOSIM values (R_unweighted_ = +0.30, *P* = 0.001) of the unweighted UniFrac distances. Moreover, the nasal microbiome differed across time, as shown by distinct clustering for weeks 0, 9, and 12 within Control and High Se calves (Fig 4A). There were no shifts in major bacteria between treatments, as indicated by non-significant treatment differences in weighted UniFrac distances (ANOSIM R_weighted_ = +0.009, *P* = 0.32). Treatment groups did not differ in nasal microbiome at week 0 (ANOSIM R_unweighted_ = +0.008, R_weighted_ = -0.011, both *P* > 0.10) and week 12 (ANOSIM R_unweighted_ = -0.081, R_weighted_ = -0.042, both *P* > 0.10).

The microbial diversity of less abundant bacteria taxa increased between High-Se calves during the Se-treatment period, as indicated by the unweighted UniFrac distances. Control calves caught up with High-Se calves in microbial diversity in the feedlot (Fig 4; Table 3). There were shifts in major bacteria taxa across time, but not between treatments, as indicated by weighted UniFrac distances (Table 3).

**Table 3:** Effect of feeding Se-biofortified hay in a 9-week preconditioning period on nasal microbiome diversity within treatment groups.

Βeta-diversity indicators*	Control	High Se	SEM	*P* value
UniFrac distances	(n = 15)	(n = 15)		
**Unweighted**				
Week 0 (Weaning)	0.503^c^	0.531^b^	0.009	0.03
Week 9 (End of Se Treatment)	0.478^d^	0.582^a^	0.011	<0.0001
Week 12 (Feedlot)	0.582^a^	0.578^a^	0.004	0.49
**Weighted**				
Week 0 (Weaning)	0.231^b^	0.232^b^	0.021	0.96
Week 9 (End of Se Treatment)	0.210^b^	0.226^b^	0.017	0.51
Week 12 (Feedlot)	0.303^a^	0.333^a^	0.018	0.25

*Results are presented as least-squared means and pooled standard errors of the mean. LSMeans with different superscripts (a,b,c,d) differ across treatment and time at *P* < 0.01.

The nasal microbiome changed during the preconditioning period in all calves, with greater changes in less abundant bacteria taxa observed in High-Se calves compared with Control calves (Table 4). Both the transition from weaning (week 0) to the Se-supplementation preconditioning period (week 9), and the transition from the preconditioning period (week 9) to the feedlot (week 12) promoted changes in nasal microbiome, but the largest differences were observed between weaning (week 0) and feedlot (week 12) for both groups of calves.

**Table 4:** Effect of feeding Se-biofortified hay in a 9-week preconditioning period on nasal microbiome diversity across time.

Βeta-diversity indicators*	Control	High Se	SEM	*P* value
UniFrac distances	(n = 15)	(n = 15)		
**Unweighted**				
Week 0 vs 9	0.607^d^	0.697^bc^	0.015	0.0002
Week 9 vs 12	0.660^c^	0.652^c^	0.009	0.58
Week 0 vs 12	0.722^ab^	0.730^a^	0.012	0.62
**Weighted**				
Week 0 vs 9	0.273^b^	0.279^b^	0.024	0.86
Week 9 vs 12	0.343^ab^	0.400^a^	0.026	0.14
Week 0 vs 12	0.360^ab^	0.386^ab^	0.031	0.56

*Results are presented as least-squared means and pooled standard errors of the mean. LSMeans with different superscripts (a,b,c,d) differ across treatment and time at *P* < 0.01.

### Effect of feeding weaned beef calves Se-biofortified alfalfa hay in a preconditioning program on nasal microbiota

**Fig 5:**
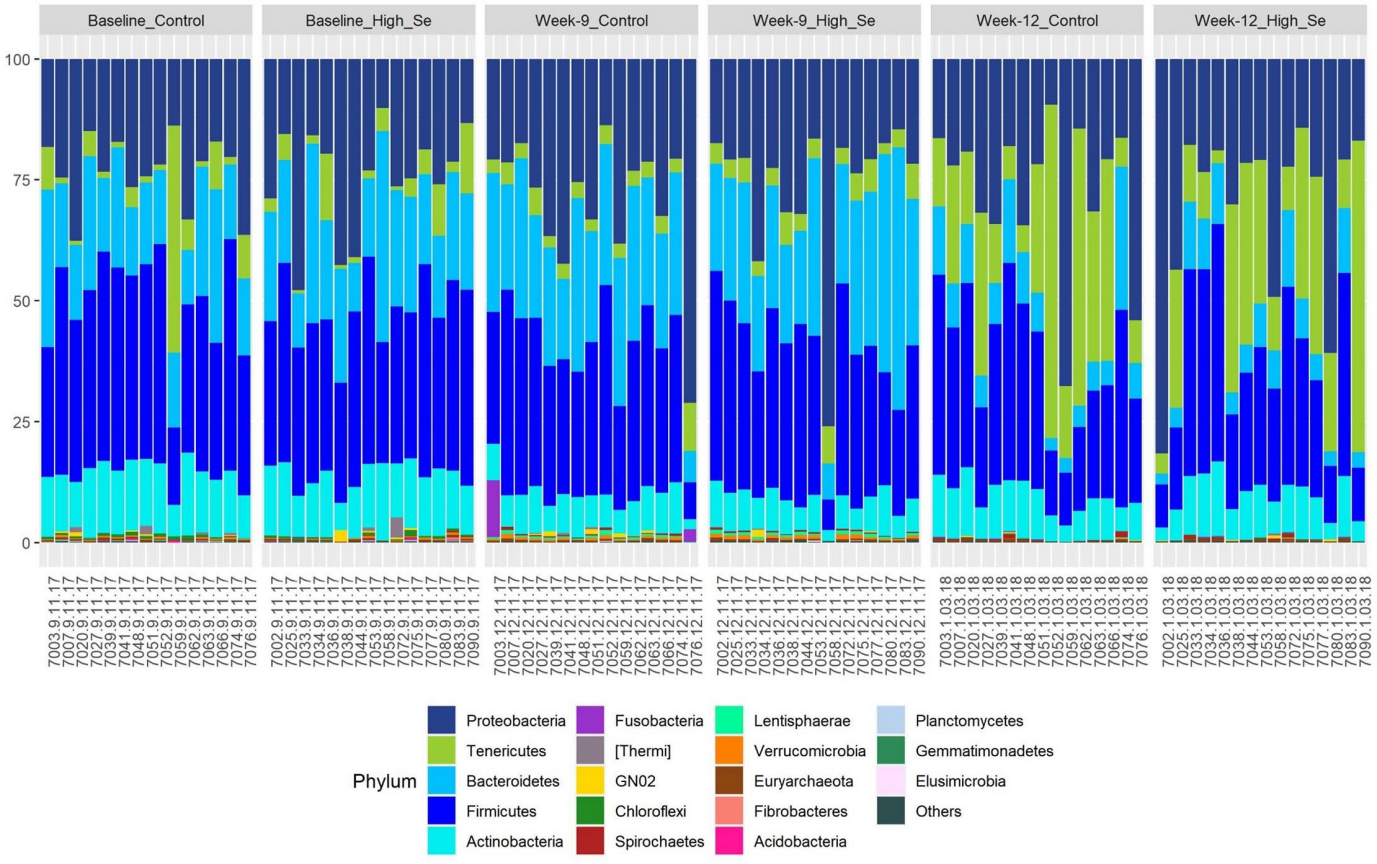
Nasal microbiota profiles of healthy calves after weaning (week 0), at the end of the Se-supplementation preconditioning period (week 9), and in the feedlot (week 12). During the 9 week preconditioning period, calves consumed alfalfa hay harvested from a field not fertilized with Se (Control) or harvested from a field fertilized with sodium-selenate (High Se; application rate of 90 g Se/ha; n = 15 calves per group for Control and High Se).

We detected 23 bacterial phyla in nasal swabs across time and treatment. The profiles of the 19 most abundant bacterial phyla for individual calves across time are shown in Fig 5 and show large changes in major phyla across time. The proportion of the phylum *Tenericutes*, which contains genera of the *Mycoplasmataceae* family, increased dramatically after calves entered the feedlot. Some calves had over 50% *Tenericutes*. The phylum most negatively impacted by the increase in *Tenericutes* was *Bacteroidetes*.

The number of nasal microbiota phyla within High-Se calves increased during the Se-treatment period (Table 5). Five bacterial phyla were present only in High-Se calves, which were *Acidobacteria*, *Chlamydiae*, *OP11*, *TM7*, and *WPS-2*. Control calves caught up with High-Se calves in phyla diversity after they entered the feedlot; however, *Chlamydiae*, *TM7*, and *WPS-2* remained present only in High-Se calves.

**Table 5:** Effect of feeding Se-biofortified hay in a 9-week preconditioning period on number of nasal microbiota phyla.

	Bacterial Phyla (number)*			
Time Period	Control	High Se	SEM	*P* value
	(n = 15)	(n = 15)		
Week 0 (Weaning)	13.60^b^	13.00^b^	0.57	0.46
Week 9 (End of Se Treatment)	13.40^b^	18.93^a^	0.72	<0.0001
Week 12 (Feedlot)	16.40^a^	17.13^a^	0.43	0.24

*Results are presented as least-squared means and pooled standard errors of the mean. LSMeans with different superscripts (a,b) differ across treatment and time at *P* < 0.01.

For the statistical analysis, we partitioned bacterial phyla into those that were detected in at least 14 of 15 calves per group across treatment and time (Table 6) and those that were detected only in a subset of calves (Table 7). Bacterial taxa present in Control and High-Se treatment calves at ≥ 1% mean relative abundance in Control calves are shown in S1 Table. There were minor effects of feeding Se-biofortified hay on the consistently present phyla, which were present in descending order of prevalence as follows: *Firmicutes*, *Proteobacteria*, *Bacteroidetes*, *Actinobacteria*, *Tenericutes*, *Euryarchaeota*, and *Verrucomicrobia* (Table 6). The proportion of *Tenericutes* increased from weaning to the feedlot, and was accelerated by feeding Se-biofortified hay in the preconditioning period. There was a statistical trend (*P* = 0.08) for higher proportions of *Verrucomicrobia* after feeding Se-biofortified hay in the preconditioning period.

**Table 6:** Effect of feeding Se-biofortified hay in a 9-week preconditioning period on relative abundance, median % (interquartile range), of the major nasal microbiota phyla*.

Phylum	Control	High Se	*P* value
	(n = 15)	(n = 15)	
***Firmicutes***			
Week 0 (Weaning)	36.8 (28.9, 42.9)	32.4 (30.2, 40.6)	0.42
Week 9 (End of Se Treatment)	31.7 (27.2, 36.6)	32.5 (26.1, 37.9)	0.69
Week 12 (Feedlot)	32.5 (20.7, 38.1)	24.5 (16.9, 42.0)	0.98
***Proteobacteria***			
Week 0 (Weaning)	21.8 (17.1, 26.5)	23.1 (15.8, 28.8)	0.79
Week 9 (End of Se Treatment)	25.5 (20.8, 36.7)	20.8 (17.5, 31.7)	0.19
Week 12 (Feedlot)	21.8 (16.4, 34.2)	22.3 (18.9, 43.5)	0.58
***Bacteroidetes***			
Week 0 (Weaning)	16.0 (15.4, 26.8)	21.3 (17.0, 23.9)	0.31
Week 9 (End of Se Treatment)	26.4 (21.8, 30.6)	25.4 (20.3, 31.8)	0.72
Week 12 (Feedlot)	8.0 (5.0, 12.1)	7.9 (4.1, 12.5)	0.58
***Actinobacteria***			
Week 0 (Weaning)	12.9 (11.7, 14.5)	12.2 (10.6, 14.4)	0.60
Week 9 (End of Se Treatment)	7.5 (6.4, 8.4)	7.0 (4.7, 8.4)	0.58
Week 12 (Feedlot)	8.7 (6.2, 11.1)	9.8 (6.3, 12.1)	0.92
***Tenericutes***			
Week 0 (Weaning)	1.52 (1.14, 8.86)	2.81 (1.25, 5.34)	0.88
Week 9 (End of Se Treatment)	3.13 (2.91, 3.95)	4.11 (3.47, 6.75)	0.04
Week 12 (Feedlot)	15.0 (8.80, 33.6)	20.3 (9.61, 36.7)	0.92
***Euryarchaeota***			
Week 0 (Weaning)	0.30 (0.27, 0.43)	0.29 (0.22, 0.41)	0.58
Week 9 (End of Se Treatment)	0.37 (0.20, 0.44)	0.34 (0.20, 0.40)	0.63
Week 12 (Feedlot)	0.31 (0.14, 0.42)	0.30 (0.12, 0.44)	0.95
***Verrucomicrobia***			
Week 0 (Weaning)	0.25 (0.19, 0.33)	0.20 (0.12, 0.32)	0.49
Week 9 (End of Se Treatment)	0.40 (0.26, 0.50)	0.63 (0.38, 0.78)	0.08
Week 12 (Feedlot)	0.03 (0.02, 0.04)	0.03 (0.02, 0.04)	0.66

*Phyla that were observed in 14 or more of 15 calves of Control and High-Se groups throughout the production stages are presented as median % (25%, 75%) relative abundance. Kruskal Wallis rank test was used to compare treatment groups.

**Table 7:** Effect of feeding Se-biofortified hay in a 9-week preconditioning period on the presence, and number of calves harboring per Control (C) and High-Se (Se) treatment groups, of the minor nasal microbiota phyla*.

	Week 0		Week 9		Week 12		*P* values		
Phylum	C	Se	C	Se	C	Se	Wk 0	Wk 9	Wk 12
	(n = 15)	(n = 15)	(n = 15)	(n = 15)	(n = 15)	(n = 15)			
*Acidobacteria*	13	12	0	7	2	3	1	0.002	1
*Chlamydiae*	0	0	0	9	0	3	1	0.0007	0.22
*Chloroflexi*	15	13	10	13	15	15	0.48	0.39	1
*Elusimicrobia*	2	1	11	14	7	8	1	0.33	1
*Fibrobacteres*	11	8	9	12	12	13	0.45	0.43	1
*Fusobacteria*	2	2	6	10	14	14	1	0.27	1
*GN02*	9	6	9	10	12	11	0.17	1	1
*Gemmatimonadetes*	10	11	1	8	12	9	1	0.01	0.43
*Lentisphaerae*	4	9	15	15	14	12	0.14	1	0.60
*OP11*	0	1	0	8	0	0	1	0.002	1
*Planctomycetes*	12	11	6	11	10	11	1	0.14	1
*Spirochaetes*	12	8	14	15	15	15	0.25	1	1
*TM7*	0	0	0	11	0	6	1	<0.0001	0.02
*WPS-2*	0	1	0	9	0	1	1	0.0007	1
*Thermi*	10	9	14	15	15	15	1	1	1
*Other Bacteria*	1	0	2	12	13	13	1	0.0007	1

*Phyla that were observed in 13 calves or fewer of Control and High-Se calves throughout the production stages (week 0 = weaning; week 9 = end of Se supplementation preconditioning period; week 12 = feedlot period) are presented as number of calves where phyla are present. Fisher’s Exact test was used to compare treatment groups at each time (0, 9, and 12 weeks).

The major effects of feeding Se-biofortified hay was on the minor bacterial phyla (Table 7). Feeding Se-biofortified hay promoted the presence of minor bacterial phyla. Five bacterial phyla: *Acidobacteria*, *Chlamydiae*, *OP11*, *TM7*, and *WPS-2*, were only present in High-Se calves in week 9. In addition, *Gemmatimonadetes* and *Other Bacteria* were present in more High-Se than Control calves.

We further queried on the genera level and detected a total of 322 bacterial genera in nasal swabs. The number of nasal microbiota genera within High-Se calves increased during the Se-treatment period (Table 8). A total of 56 bacterial genera (13 *Bacteroidetes* genera; 12 *Firmicutes* genera, 11 *Proteobacteria* genera, 9 *Actinobacteria*, 4 *Verrucomicrobia* genera, 2 *Thermi* genera, and one each for *Chlamydiae*, *Fibrobacteres*, *Tenericutes*, *TM7* and *WPS-2*) were present only in High-Se calves in week 9. In contrast, 10 bacterial genera were present only in Control calves in week 9. Control calves caught up with High-Se calves in genera diversity after they entered the feedlot (Table 8); however, 31 of the 56 bacterial genera (8 *Bacteroidetes* genera; 4 *Firmicutes* genera, 7 *Proteobacteria* genera, 4 *Actinobacteria*, 3 *Verrucomicrobia* genera, 2 *Thermi* genera, and one each for *Fibrobacteres*, *TM7* and *WPS-2*) remained present only in High-Se calves.

**Table 8:** Effect of feeding Se-biofortified hay in a 9-week preconditioning period on number of nasal microbiota genera.

	Bacterial Genera (number)*			
Time Period	Control	High Se	SEM	*P* value
	(n = 15)	(n = 15)		
Week 0 (Weaning)	153^b^	145^b^	8	0.46
Week 9 (End of Se Treatment)	167^b^	215^a^	9	<0.0001
Week 12 (Feedlot)	220^a^	227^a^	5	0.24

*Results are presented as least-squared means and pooled standard errors of the mean. LSMeans with different superscripts (a,b) differ across treatment and time (0, 9, and 12 weeks) at *P* < 0.01

**Fig 6:**
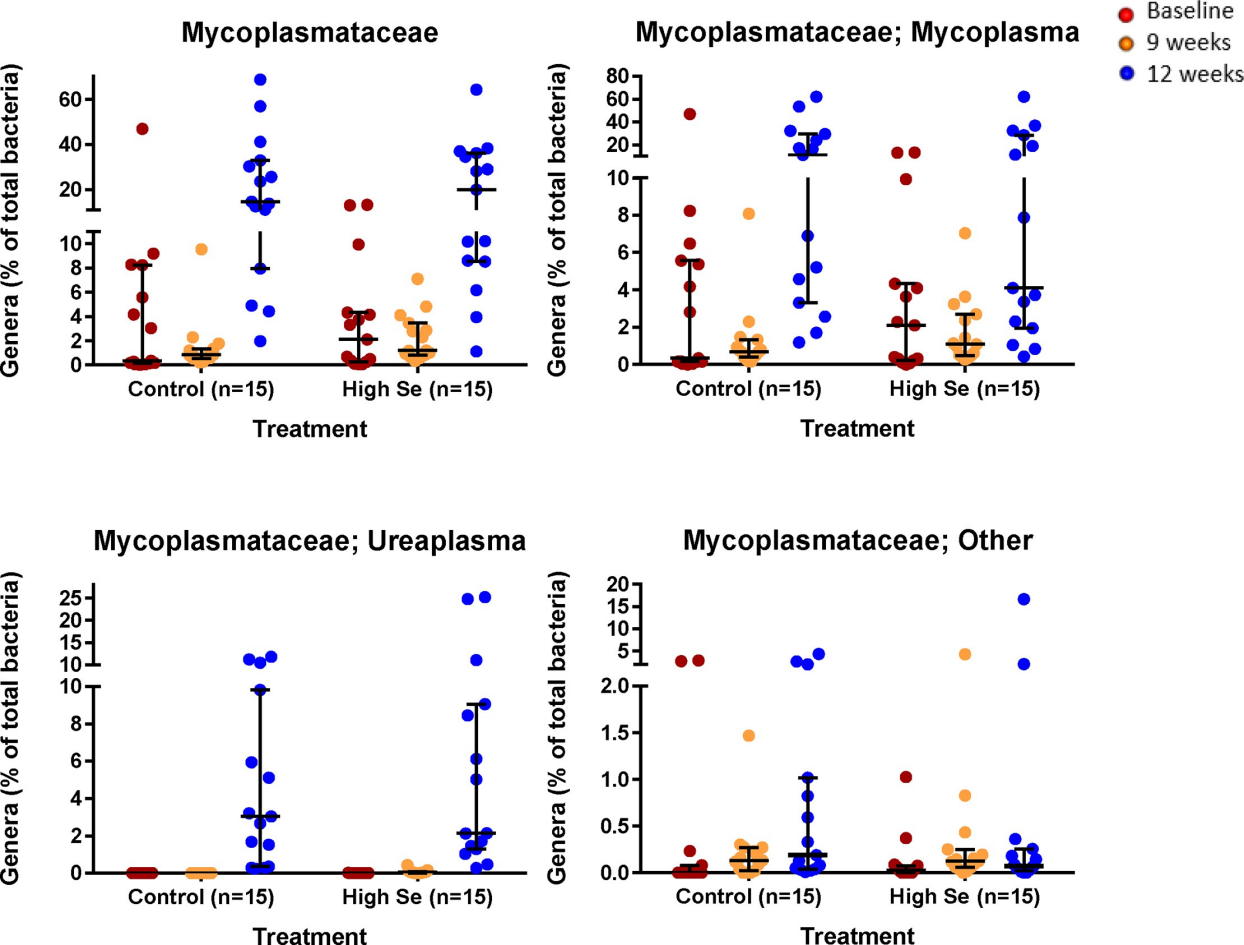
Nasal *Mycoplasmataceae* profile of healthy calves after weaning (week 0), at the end of the Se-supplementation preconditioning period (week 9), and in the feedlot (week 12). During the 9 week preconditioning period, calves consumed alfalfa hay harvested from a field not fertilized with Se (Control) or harvested from a field fertilized with sodium-selenate (High Se; application rate of 90 g Se/ha; n = 15 calves per group for Control and High Se).

Eighteen bacterial genera were present only in High-Se calves in week 9, and in both treatment groups in the feedlot: 5 *Bacteroidetes* genera; 7 *Firmicutes* genera, 3 *Proteobacteria* genera, 2 *Actinomycetales* genera, and one *Tenericutes* genus. The Tenericutes genus *Ureaplasma* of the *Mycoplasmataceae* family was especially intriguing (Fig 6). At weaning (week 0), *Ureaplasma* was only detectable at low proportions in one High-Se calf. In week 9, 10 High-Se calves and no Control calves had *Ureaplasma* (*P* = 0.0002). In the feedlot, all calves had *Ureaplasma* and the proportions had dramatically increased: *Ureaplasma* was the sixth most abundant bacterial genus in Control calves and the third most abundant genus in High-Se calves with no treatment differences observed.

All three detected genera of the *Mycoplasmataceae* family (genus *Mycoplasma*, *Ureaplasma*, and *Other Mycoplasmataceae*) increased dramatically after calves entered the feedlot (Fig 6). Similar changes were not observed in the other 5 detected genera of the phylum *Tenericutes* (results not shown). In the feedlot, *Mycoplasma* was the most abundant bacterial genus in both treatment groups with some calves having over 50% of their nasal microbiota being *Mycoplasma* (Fig 6). In contrast to *Ureaplasma*, some calves had at weeks 0 and 9 high proportions of *Mycoplasma* and *Other Mycoplasmataceae* already at weeks 0 and 9 (Fig 6). Furthermore, proportions of *Mycoplasma* and *Other Mycoplasmataceae* were not significantly altered by feeding Se-biofortified hay.

### Effect of feeding weaned beef calves Se-biofortified alfalfa hay in a preconditioning program on nasal functional microbiome

We detected a total of 452 different predicted metabolic pathways in nasal swabs using PICRUSt2. The effect of feeding weaned beef calves Se-biofortified alfalfa hay was smaller on nasal microbial metabolism compared with its effect on nasal microbiota or total microbiome. There was a minor increase in the number of predicted metabolic pathways in response to feeding Se-biofortified hay (Table 9). Control calves caught up with High-Se calves in the number of predicted metabolic pathways in the feedlot (Table 9).

**Table 9:** Effect of feeding Se-biofortified hay in a 9-week preconditioning period on number of predicted metabolic pathways in nasal microbiota.

	Predicted Metabolic Pathways (number)*			
Time Period	Control	High Se	SEM	*P* value
	(n = 15)	(n = 15)		
Week 0 (Weaning)	391^b^	386^b^	2.7	0.16
Week 9 (End of Se Treatment)	387^b^	399^a^	2.8	0.004
Week 12 (Feedlot)	404^a^	404^a^	1.8	0.82

*Results are presented as least-squared means and pooled standard errors of the mean. LSMeans with different superscripts (a,b) differ across treatment and time (0, 9, and 12 weeks) at *P* < 0.01.

Pathways present in Control and High-Se treatment calves at ≥ 1% mean relative abundance in Control calves are shown in S2 Table. The primary effect of feeding Se-biofortified alfalfa hay was in less abundant predicted metabolic pathways. Eight predicted metabolic pathways were only present in High-Se calves in week 9, whereas no predicted metabolic pathway was only present in Control calves (results not shown). Thirteen predicted metabolic pathways were present in greater numbers of High-Se calves compared with Control calves (week 9), whereas no predicted metabolic pathways were present in greater numbers of Control calves compared with High-Se calves. Of the remaining five significantly different predicted metabolic pathways, two were higher in High-Se calves compared with Control calves and three were lower.

**Fig 7:**
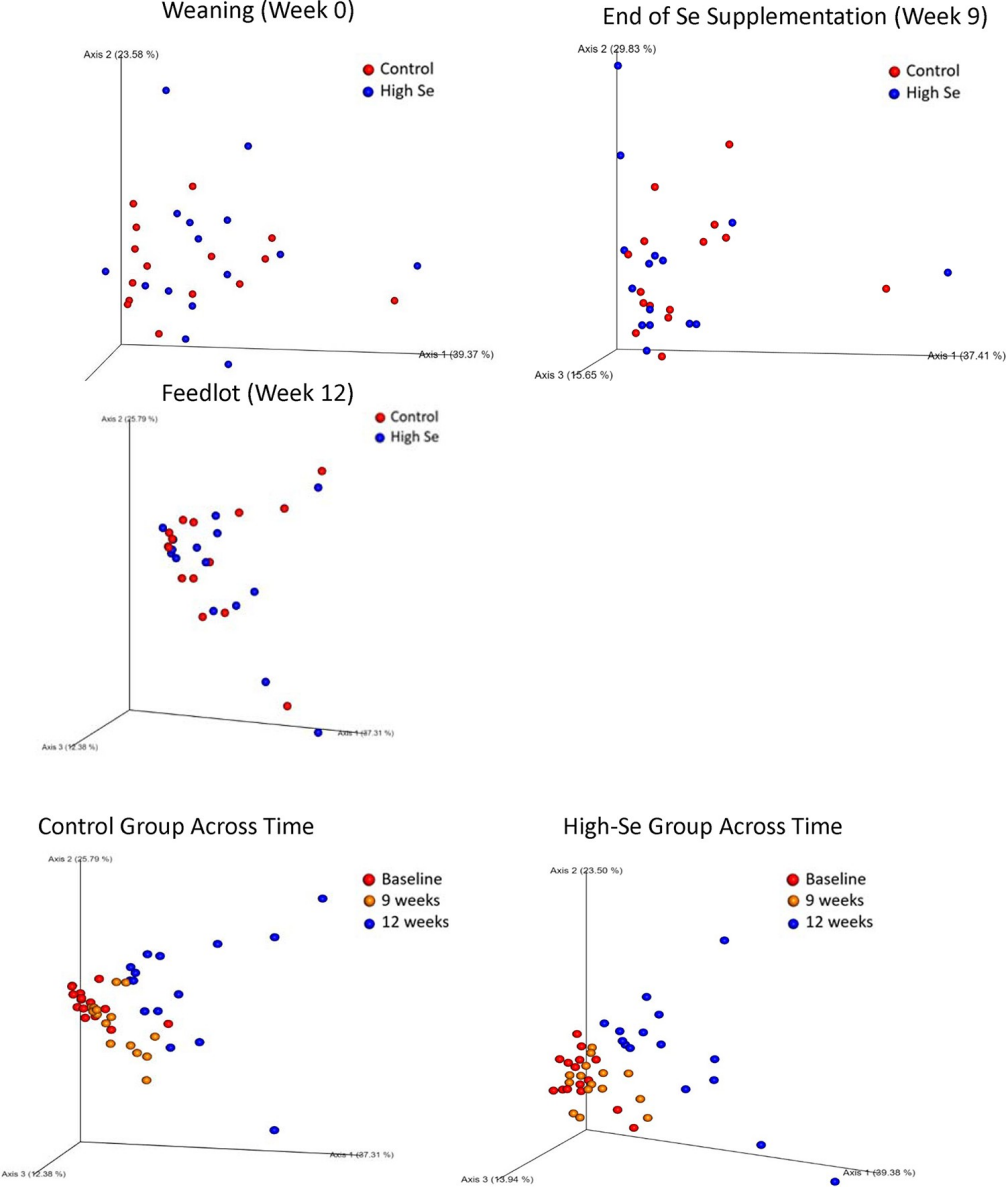
Principal coordinate analysis of Bray-Curtis distances of predicted metabolic pathways for healthy calves after weaning (week 0), at the end of the Se-supplementation preconditioning period (week 9), and in the feedlot (week 12). During the 9 week preconditioning period, calves consumed alfalfa hay harvested from a field not fertilized with Se (Control) or harvested from a field fertilized with sodium-selenate (High Se; application rate of 90 g Se/ha; n = 15 calves per group for Control and High Se).

The principal coordinates of Bray Curtis Dissimilarities for predicted metabolic pathways did not differ between Control and High-Se calves at weeks 0, 9, and 12 (Fig 7). The ANOSIM values for treatment differences were R = -0.02 (*P* = 0.60) and R = -0.02 (*P* = 0.71) at weeks 0 and 9, respectively. There was a significant effect of time on nasal microbial metabolism, as indicated by PCoA plots of Bray Curtis Dissimilarities (Fig 7) and pairwise ANOSIM tests of Bray Curtis Dissimilarities across times (all *P* = 0.001; results not shown).

## Discussion

Fertilizing alfalfa with sodium selenite at 90 g Se/ha increased the Se content of alfalfa hay over 50 fold. Feeding this Se-biofortified alfalfa hay to calves during the preconditioning period increased WB-Se concentrations almost 300%, improved growth and carcass weights, increased overall scores for slaughter yield grade, and enriched the nasal microbial diversity. There was no effect on parasite load because fecal egg counts remained low throughout.

### Selenium intake

We have previously shown a linear relationship between Se fertilizer application rate and forage Se concentration [5, 6, 33, 34]. Our results suggest that Se application rate is the primary determinant of Se content for common forage species. Fertilizing the alfalfa hay field with sodium selenite at 90 g Se/ha resulted in Se content of alfalfa in this study of 3.47 mg Se/kg DM, which was within our previous range of 3.26 mg Se/kg DM [6] to 5.17 mg Se/kg DM [35] at this fertilization rate. This is more than the nutritional requirements of Se in cattle, which are conservatively estimated at 0.1 mg/kg DM [36]. At 3.47 mg Se/kg DM forage Se concentration, calculated Se intake was 26.8 mg Se/calf per day, and represented 98% of the total dietary Se intake. Compared with controls, calves consumed 25× more organic Se with agronomic biofortification. Selenomethionine is the major selenocompound in forage legumes made from Se taken up from the soil [37].

### Selenium status

Blood Se concentrations were increasing throughout the 9-week preconditioning period, and remained high relative to control calves after three weeks in the feedlot. These results are consistent with our previous studies. Consumption of Se-biofortified alfalfa hay in this study resulted in WB-Se concentrations after 9 weeks of 556 ± 11 ng/mL, which was consistent with our previous range of 278 ± 7 ng/mL (7-weeks preconditioning period) [6] to 494 ± 11 ng/mL (8 weeks preconditioning period) [35] when fed Se-biofortified alfalfa hay for shorter time periods. An advantage of feeding Se-biofortified forage is that all calves consume the Se treatment such that variation in WB-Se concentrations between calves is small.

### Growth characteristics and carcass data

Consumption of Se-fertilized alfalfa hay resulted in increased BW in weaned beef calves and tended to increase hot carcass weight (*P* = 0.07), similar to what we previously reported [6, 9]. In this study, similar to our previous study [9], carcass quality grade levels were not different between control and Se-treatment groups of calves, likely because most calves were choice grade. However yield grades in this study were improved in calves fed Se-biofortified alfalfa hay, unlike in our previous study [9]. More calves in the Se-treatment group (86%) had a 1 (most desirable trim) or 2 (industry average) compared with calves in the control group (36%). Our results contrast to those reported by Swecker et al. [38] whereby parenteral Se and vitamin E supplementation of weaned beef calves at 0 and 28 days did not affect calf performance during the 42-day preconditioning period. It is likely that the impact and results of Se supplementation depend on Se status of calves (deficient vs adequate), type (organic vs inorganic) and amount of Se administered, and route of Se administration (oral vs injectable) [39].

In this study, third cutting alfalfa hay was enriched with Se by mixing inorganic sodium-selenate with water and spraying it onto the soil and foliage of an alfalfa field at an application rate of 90 g Se/ha. In our previous study [6, 9], using similarly enriched second cutting alfalfa hay, alfalfa yield was approximately 4490 kg/ha. Each calf consumed an average of 227 kg of alfalfa during the preconditioning period. We calculated the commercial cost for application of 90 g Se/ha using Selcote Ultra^®^ (10 g Se/kg as 1:3 sodium and barium selenate, respectively) to an alfalfa field as $158/ha; thus a calculated cost of $8/head for the Se enriched alfalfa hay consumed during the preconditioning period. Market prices for slaughter calves are currently approximately $3.54/kg hot carcass weight. Applying these market prices to the gain measured in our former study, feeding calves high Se enriched alfalfa hay resulted in an increase of $166/head over that of the control group of calves. In the current study, at the end of the feedlot period, calves in the high Se group had a 9 kg higher slaughter weight compared with control calves resulting in an increase of $32/head over that of control calves. Thus, it is economically justified to recommend feeding Se-biofortified alfalfa hay during the preconditioning period to beef calves.

### Fecal parasite counts

Although fecal parasite counts were assessed at baseline and weeks 5 and 9 of the preconditioning period, and again 3 weeks after entering the feedlot, no significant treatment effects were observed. This was likely because fecal parasite counts were very low throughout the preconditioning period, and essentially zero 3 weeks after entering the feedlot because all calves were dewormed at entry to the feedlot. We had previously shown in sheep that supranutritional Se-yeast supplementation may enhance resistance to naturally occurring *H*. *contortus* gastrointestinal parasites [40]. Younger ruminants are reported to have immunological hyporesponsiveness and, thus, lower resistance to infections disease, which in calves manifests as respiratory and intestinal infections (viral and bacterial) [41]. In this study, because fecal egg counts remained low throughout, we were unable to test the effect of Se treatment on gastrointestinal parasite infestations in these calves.

### Nasal microbiome

The microbiome refers to the collection of genomes from all the microorganisms in the nasopharyngeal environment [42]. In the present study, the nasal microbiome was similar at weaning for Control calves and calves slated for High-Se treatment, as one would expect given that calves from both groups came from the same ranch. During the 9-week preconditioning period, dietary Se-biofortification diversified the nasal microbiome by enriching the number of less abundant nasopharyngeal microbial genomes, evidenced by significant differences in observed ASVs and Chao1 indices. The observed ASVs represent the number of true RNA sequences present in the nasopharynx of each calf and Chao1 is a nonparametric estimate of the minimal number of RNA sequences present [28, 29]. Thus, ASV and Chao1 estimate the number of different RNA sequences (richness). The Shannon index is also a measure of alpha diversity. It takes into account not only the number of different RNA sequences, but also the relative abundance of the different RNA sequences (evenness) [29]. Evenness compares the similarity of the population size of each of the species present. Shannon index is significant when both the number of RNA sequences increase as well as the proportion of the major sequences become more equal. Neither High-Se treatment nor transition to the feedlot increased Shannon index, i.e., evenness in our study. Thus, High-Se treatment resulted in a higher number of different RNA sequences, but did not make the different RNA sequences more similar in amounts. We previously reported [35] that increasing Se-status during a preconditioning period resulted in treatment group difference in nasal microbiota. The novel contribution of the present study is that Se-biofortification promoted diversification of the nasal microbiome by allowing the establishment of minor phyla (5 new phyla) and genera (56 new genera) and their unique metabolism pathways.

Beta diversity measured using the phylogeny-based unweighted and weighted UniFrac distances, showed that calves receiving the High-Se-treatment clustered separately from Control calves, although clustering was not significant for weighted UniFrac distances. Beta diversity measures differences between nasopharyngeal samples/calves [31]. Unweighted UniFrac distance measures differences in number of ASVs present, whereas the weighted UniFrac distance measures not only differences in number of ASVs present, but also relative abundance of the ASVs [30]. UniFrac distances measure the proportion of a phylogenetic tree that is not shared between two measures [32]. The more unshared the genome, the bigger the differences. Principal coordinate analysis calculates distances between all the samples and displays the distances in a 2 or 3 dimensional space [31]. Diversity of the less abundant bacteria increased between Control and High-Se calves during the Se-treatment period as indicated by the significantly different unweighted UniFrac distances. Control calves caught up with the High-Se calves in genomic diversity in the feedlot. None-the-less the largest differences were observed between weaning and feedlot in both groups of calves.

Several studies have examined how some management practices decrease diversity within the nasopharyngeal bacterial communities and reported that weaning, transportation methods, on-arrival processing at the feedlot, time in the feedlot [43] and antimicrobial usage [44] decrease nasal microbiome diversity. Those results suggest that stressful events may decrease diversity within nasopharyngeal bacterial communities. However, diversity changes in nasal microbiome differs amongst operations, groups, and time points (spring processing, arrival at feedlot, 40 days in the feedlot), suggesting that the respiratory microbiota of calves may lack a common pattern of evolution from ranch to feedlot [45]. The results in the present study are in line with those findings, as we observed a change over time (weaning, preconditioning period, and feedlot) in nasal microbiome diversity, which differed depending on Se-biofortification.

Another important factor that decreases nasal microbiome diversity is respiratory health status. Alpha-diversity indices revealed a lower bacterial diversity in the nasopharynx of steers with bronchopneumonia compared to healthy pen-mates [46, 47]. Bacterial communities in the nasopharynx of cattle that remained healthy during the feeding period were more diverse than those that developed BRD [1], suggesting that management practices that increase microbial diversity may aid in prevention of respiratory diseases in the feedlot, which are especially common on entry to the feedlot. We have shown twice now that High-Se treatment of calves in a preconditioning program was able to upregulate the diversity of the nasopharyngeal bacterial community prior to entering the feedlot, which may ameliorate a risk factor for development of BRD [35]. To date, we are not aware of other management practices that increase diversity of the nasopharyngeal bacterial community, and thereby, potentially improve health status.

The question arises how increasing the diversity of the nasopharyngeal bacterial community improves health status. Microbial biodiversity correlates to the efficiency of nutrient utilization by a bacterial community; a greater microbial diversity means greater diversity in microbial metabolism pathways, which allows for more efficient nutrient utilization. This, in turn, decreases the chances of bacterial respiratory pathogens adhering to and colonizing in the nasopharynx (reviewed in [46]). This becomes especially important when stressed calves enter the feedlot, and are exposed to a variety of new pathogens.

There is limited information how dietary Se may increase the diversity of the nasopharyngeal bacterial community. We have reported over the last decade that Se-supplementation to Se-replete livestock can prevent infectious diseases [8–10, 40, 48, 49]. We proposed that the mechanism by Se prevents infectious diseases is through selenoproteins, which improve the functional immune status of livestock [5, 9, 10, 34, 48, 49]. The results of this study suggest a potential interplay between the immune system of the host and its nasopharyngeal bacterial community. We hypothesize that Se-biofortification may alter nose-associated lymphoid tissues (NALT), which include T cells, B cells, dendritic cells, and macrophages [50, 51], which in turn alters nasopharyngeal bacterial communities. This mechanism has been proposed as one role the immune system plays in regulating the microbiota [52]. Alternatively, Se-biofortification may alter the amount and composition of the nasal mucus layer, which is an important nutrient source for the nasopharyngeal bacterial community [42, 52, 53]. These are future directions of study for our research.

### Nasal microbiota

The microbiota refers to specific microorganisms found within the nasopharynx [42]. At baseline (weaning) the major nasopharynx phyla (with relative abundance >1%) were *Firmicutes* (35%), *Proteobacteria* (22%), *Bacteroidetes* (19%), and *Actinobacteria* (13%), and *Tenericutes* (2%). These were similar for Control calves and calves slated for High-Se treatment. We did not have baseline microbiota data in our former study [35], but in Control calves at 8 weeks, the majority of sequences were classified as *Proteobacteria* (44%). The predominant bacterial phyla in the current study were similar to those reported by others for calves at entry to the feedlot [54], although there is variation in phyla and genera among studies as evidenced by *Tenericutes* ranging from 47.4% [46] to 22.5% [47] to 11.2% [54] in different studies. We were specifically interested in the phyla *Tenericutes*, as *Tenericutes* was the only phylum with relative abundance >1% altered by Se-biofortification in our current study and in our previous study [35].

In the present study, the class *Mollicutes* of the phylum *Tenericutes* was the only bacterial class significantly altered by Se-biofortification. *Mollicutes* are unique in that they lack a cell wall and include the smallest bacterial species with the shortest genome sequences; as a result, *Mollicutes* species adhere to and sometimes enter nasal epithelial cells and rely heavily on nutrients from the host (reviewed by Parker et al. [55]). *Mollicutes* bacteria develop a close interaction with the host and employ various mechanisms to evade elimination by both the innate and adaptive immune systems, which makes treatment more challenging [56].

Of the 7 known *Mollicutes* genera, time and treatment affected primarily the 3 genera of the family *Mycoplasmataceae*: *Ureaplasma*, *Mycoplasma*, and *Other Mycoplasmataceae*, all of which increased dramatically after calves entered the feedlot. In the feedlot, the predominant genera in the present study were unclassified (24%), *Mycoplasma* (16%), *Moraxella* 6%, and *Ureaplasma* (6%). We did not see high numbers of *Acinobacter* as reported by others [54]. A similar increase in the family *Mycoplasmataceae* has been reported previously after calves entered the feedlot [43], which can be prevented by antibiotic treatment with macrolides that interfere with microbial RNA replication [44]. Unique to the present study was the dramatic increase in *Ureaplasma* after calves entered the feedlot and the effect of Se-biofortification on the presence of *Ureaplasma* during the preconditioning period.

*Ureaplasma* and *Mycoplasma* are very similar in structure and function, and both are linked to a variety of inflammatory diseases in cattle; their differences are in their colony formation structure, the fact that only *Ureaplasma* can secrete urease, and in the number of species they contain [57, 58]. In contrast to over 120 identified *Mycoplasma* species, there are only 11 identified bovine *Ureaplasma* species, the best known species being *Ureaplasma parvum*, which is prevalent in many herds and linked to a high incidence of abortions (i.e., bovine ureaplasmosis) [57, 59, 60]. Distinct bacterial metacommunities inhabit the upper and lower respiratory tracts of healthy feedlot cattle and those diagnosed with bronchopneumonia [61]. *Ureaplasma parvum* has been identified in the nose and the lung of cattle and its increased presence has been linked with *Mycoplasma* to BRD [46, 62, 63]. Similar to *Mycoplasma* species, *Ureaplasma* species are usually benign; however, their ability to secrete toxins and induce a pro-inflammatory response in the host can cause extensive epithelial tissue damage [64, 65]. Moreover, a large number of *Ureaplasma* or *Mycoplasma* species could decrease feed efficiency because of their nutrient dependency on the host. The question arises why *Ureaplasma* was present in 2/3 of High-Se calves, but none of the Control calves at the end of the preconditioning period. One hypothesis is that *Ureaplasma* species are able to evade detection by the immune system of the host by hiding in epithelial cells [64], which gives this bacterium an edge when invading ecological niches in hosts supplemented with immunomodulatory Se.

In the present study, *Mycoplasma* was the most abundant bacterial genus in both Control and High-Se treatment calves in the feedlot (over 50% of microbiota in some calves). In contrast to *Ureaplasma*, some Control and High-Se treatment calves had high proportions of *Mycoplasma* at baseline and 9 weeks. Others have reported a large increase in *Mycoplasma* after arrival at the feedlot [45], which they considered to be unrelated to the use of antimicrobials [47]. The genus *Mycoplasma* contains over 120 species and their differentiation is challenging [55, 66, 67]. Because *Mycoplasma* species differ in pathogenicity, polymerase chain reaction (PCR) techniques are the standard method for *Mycoplasma* diagnosis [55]. In the present study, we did not speciate *Mycoplasma* using a ASVs approach with DADA2, because assignment to specific variants are based on small differences in ASVs [61] and only differentiate a few *Mycoplasma* species [46]. We also did not speciate *Mycoplasma* by PCR techniques so we cannot further characterize the increase in *Mycoplasma* species.

Bovine respiratory disease is one of the most common causes of health and economic losses in the feedlot and closely linked to an animal’s commensal bacteria populations in the respiratory tract including *Mycoplasma* [68, 69]. Several *Mycoplasma* species are opportunistic pathogens present in symptomatic and asymptomatic cattle with *Mycoplasma bovis* being the most pathogenic [47, 55, 69]. A recent study reported a shared metacommunity between the lung and nasopharynx for *Mycoplasma* species and suggested the nasopharynx as the best target for management strategies against BRD [61], as it could prevent for example *Mycoplasma* adherence.

One interesting feature in the present study was the effect of feeding Se-biofortified hay on the number of minor phyla and genera present at the end of the Se-supplementation period, which persisted in the feedlot. After feeding Se-biofortified hay for 9 weeks, the number of phyla within High-Se calves increased compared with control calves from 13 to 19 phyla, whereas Control calves had no change in number of phyla compared with weaning (13 phyla). The increase in phyla numbers in High-Se calves was the result of increased numbers of the minor bacterial phyla (phyla observed in 13 or fewer of Control and High-Se calves). Five bacterial phyla were present only in High-Se calves at 9 weeks. Although Control calves caught up with High-Se calves in phyla diversity after they entered the feedlot (increased from 13 to 16 phyla), three of the minor bacterial phyla were still only present in the High-Se calves. Similarly, the number of microbiota genera within High-Se calves increased during the High-Se treatment period. A total of 56 bacterial genera (>90% in the major phyla) were present only after High-Se treatment, whereas only 10 genera were present solely in Control calves at 9 weeks. Again, Control calves caught up with High-Se calves in genera diversity after they entered the feedlot. None-the-less, 31 bacterial genera (>85% in the major phyla) remained present only in High-Se calves in the feedlot. Coincidently, WB-Se concentrations remained higher in High-Se treated calves after 3 weeks in the feedlot. We propose that the increased diversity of nasal microbiota present after 9 weeks of consuming Se-fortified alfalfa, and still present 3 weeks after entering the feedlot, may provide a bacterial community component for potentially regulating bacterial overgrowth and respiratory disease caused by *Mycoplasmataceae*.

### Nasal microbiome predicted metabolic pathways

The effect of feeding weaned beef calves Se-biofortified alfalfa hay was smaller on nasal microbial metabolism compared with its effect on the nasal microbiome and microbiota. There was a small increase in the number of predicted metabolic pathways in response to feeding Se-biofortified hay, but Control calves caught up with High-Se calves in the number of predicted metabolic pathways in the feedlot. Again, the primary effect of feeding Se-biofortified alfalfa hay was in the less abundant predicted metabolic pathways.

Bray Curtis dissimilarities measures dissimilarities between samples with no difference being zero and maximum differences being one; this measure only considers differences in abundances and does not account for phylogenetic tree differences [32]. Because High-Se treatment primarily affected the presence of minor phyla, the unweighted Unifrac measures better captured this effect in our study than Bray Curtis Dissimilarities. Thus, Bray Curtis Dissimilarities for predicted metabolic pathways did not differ between Control and High-Se calves at baseline, 9 or 12 weeks. However, there was a significant effect of time on nasal microbial metabolism, and Bray Curtis Dissimilarities were different across time.

These results suggest that the nasopharyngeal microbiota in weaned beef calves can be modified by feeding Se-biofortified alfalfa hay in a preconditioning period prior to entering the feedlot. The nasopharyngeal microbiota is important for overall respiratory health, especially when stressors such as weaning, transportation, and feed adaptation impair the normal respiratory defenses (reviewed in [70]). Because cattle diagnosed with respiratory disease had less bacterial diversity and fewer ASVs at feedlot entry [1], reduced diversity may be a risk factor to developing respiratory disease. Our results from this study, add to those previously reported [35] by following calves into the feedlot, and support feeding Se-biofortified alfalfa hay to weaned beef calves prior to entering the feedlot as an effective strategy for increasing nasopharyngeal microbial diversity.

## Conclusions

In summary, Se fertilization of alfalfa fields in a region with Se-deficient soils increases Se content of alfalfa hay. We have now shown in two independent trials that feeding recently weaned beef calves Se-biofortified alfalfa hay during the preconditioning period is an effective management strategy to build Se-body reserves, optimize growth prior to entering the feedlot, and improve feedlot performance as evidenced by higher slaughter yield grades. The novel finding of the present study is that feeding recently weaned beef calves Se-biofortified alfalfa hay during the preconditioning period promotes nasal microbiome and microbiota diversity, which in turn may provide health benefits by ameliorating a risk factor for BRD.

## Supporting information

S1 Table Bacterial taxa present at ≥ 1% mean relative abundance in Control calves (n = 15) and High-Se treatment calves (n = 15) after weaning (baseline; week 0), at the end of the Se-supplementation preconditioning period (week 9), and in the feedlot (week 12). Mean and median relative percentages of the most abundant bacterial groups are shown, annotated to the level of phylum, family, and genus, based on sequencing of the 16S rRNA.
(XLSX)

S2 Table Functional metabolism pathways present at ≥ 0.5% mean relative abundance in Control calves (n = 15) and High-Se treatment calves (n = 15) after weaning (baseline; week 0), at the end of the Se-supplementation preconditioning period (week 9), and in the feedlot (week 12). Mean and median relative percentages of the most abundant functional metabolism pathways based on sequencing of the 16S rRNA are shown. Bacterial taxa in bold significantly differ between treatment groups.
(XLSX)

## Abbreviations

ADF: acid detergent fiber
ANOSIM: analysis of similarity
ASV: amplicon sequence variant
BRD: bovine respiratory disease
BW: body weight
CP: crude protein
DM: dry matter
FDR: false discovery rate
ICP-MS: inductively coupled argon plasma emission spectroscopy
LDA: linear discriminant analysis
LEfSe: linear discriminant analysis effect size
NDF: neutral detergent fiber
PBS: phosphate buffered saline
PCoA: principal coordinate analysis
PCR: polymerase chain reaction
PICRUSt: phylogenetic investigation of communities by reconstruction of unobserved states
QIIME: quantitative insights into microbial ecology
Se: selenium
TSS: total sum scaling
WB: whole blood

## References

[1] Holman DB, McAllister TA, Topp E, Wright ADG, Alexander TW. The nasopharyngeal microbiota of feedlot cattle that develop bovine respiratory disease. Vet Microbiol. 2015;180(1–2):90–5. 10.1016/j.vetmic.2015.07.031 ISI:000361862200013. 26249828

[2] Timsit E, Workentine M, Schryvers AB, Holman DB, van der Meer F, Alexander TW. Evolution of the nasopharyngeal microbiota of beef cattle from weaning to 40 days after arrival at a feedlot. Vet Microbiol. 2016;187:75–81. 10.1016/j.vetmic.2016.03.020 ISI:000375170100011. 27066712

[3] Oldfield JE. Historical perspectives on selenium. Nutr Today. 2001;36:100–2.

[4] Filley SJ, Peters A, Bouska C. Effect of selenium fertilizer on forage selenium content. J Anim Sci. 2007;85:35–. ISI:000249692700108.

[5] Hall JA, Harwell AM, Van Saun RJ, Vorachek WR, Stewart WC, Galbraith ML, et al Agronomic biofortification with selenium: Effects on whole blood selenium and humoral immunity in beef cattle. Anim Feed Sci Tech. 2011;164(3–4):184–90. 10.1016/j.anifeedsci.2011.01.009 ISI:000289383900006.

[6] Hall JA, Bobe G, Hunter JK, Vorachek WR, Stewart WC, Vanegas JA, et al Effect of feeding selenium-fertilized alfalfa hay on performance of weaned beef calves. PLoS One. 2013;8(3):e58188 Epub 2013/03/29. 10.1371/journal.pone.0058188 23536788 PMC3594272

[7] He ZL, Yang XE, Stoffella PJ. Trace elements in agroecosystems and impacts on the environment. J Trace Elem Med Biol. 2005;19(2–3):125–40. Epub 2005/12/06. 10.1016/j.jtemb.2005.02.010 . 16325528

[8] Hall JA, Bailey DP, Thonstad KN, Van Saun RJ. Effect of parenteral selenium administration to sheep on prevalence and recovery from footrot. J Vet Intern Med. 2009;23(2):352–8. Epub 2009/02/05. 10.1111/j.1939-1676.2008.0253.x [pii]. . 19192142

[9] Hall JA, Bobe G, Vorachek WR, Hugejiletu, Gorman ME, Mosher WD, et al Effects of feeding selenium-enriched alfalfa hay on immunity and health of weaned beef calves. Biol Trace Elem Res. 2013;156(1–3):96–110. Epub 2013/10/22. 10.1007/s12011-013-9843-0 . 24142411

[10] Hall JA, Vorachek WR, Stewart WC, Gorman ME, Mosher WD, Pirelli GJ, et al Selenium supplementation restores innate and humoral immune responses in footrot-affected sheep. PLoS One. 2013;8(12):e82572 Epub 2013/12/18. 10.1371/journal.pone.0082572 24340044 PMC3855423

[11] MWPS-6. Beef Housing and Equipment Handbook. 4th ed. Iowa State University, Ames, IA, USA: Midwest Plan Service; 1987.

[12] Davis TZ, Stegelmeier BL, Panter KE, Cook D, Gardner DR, Hall JO. Toxicokinetics and pathology of plant-associated acute selenium toxicosis in steers. J Vet Diagn Invest. 2012;24(2):319–27. 10.1177/1040638711435407 ISI:000307114500011. 22379047

[13] AOAC. Official Methods of Analysis. 17th ed. Arlington, VA, USA: Association of Official Analytical Chemists; 2000.

[14] Van Soest PJ, Robertson JB, Lewis BA. Methods for dietary fiber, neutral detergent fiber, and nonstarch polysaccharides in relation to animal nutrition. J Dairy Sci. 1991;74(10):3583–97. Epub 1991/10/01. 10.3168/jds.S0022-0302(91)78551-2 . 1660498

[15] Krishnamoorthy U, Muscato TV, Sniffen CJ, Vansoest PJ. Nitrogen fractions in selected feedstuffs. J Dairy Sci. 1982;65(2):217–25. 10.3168/jds.S0022-0302(82)82180-2 ISI:A1982NG27200008.

[16] Whitlock HV. Some modifications of the McMaster helminth egg-counting technique and apparatus. J Counc Sci Ind Res (Australia). 1948;21:177–80.

[17] Jurasek ME, Bishop-Stewart JK, Storey BE, Kaplan RM, Kent ML. Modification and further evaluation of a fluorescein-labeled peanut agglutinin test for identification of Haemonchus contortus eggs. Vet Parasitol. 2010;169(1–2):209–13. Epub 2010/01/12. 10.1016/j.vetpar.2009.12.003 . 20060646

[18] Cebra CK, Stang BV. Comparison of methods to detect gastrointestinal parasites in llamas and alpacas. J Am Vet Med Assoc. 2008;232(5):733–41. Epub 2008/03/04. 10.2460/javma.232.5.733 . 18312182

[19] Bolyen E, Rideout JR, Dillon MR, Bokulich NA, Abnet CC, Al-Ghalith GA, et al Reproducible, interactive, scalable and extensible microbiome data science using QIIME 2. Nat Biotechnol. 2019;37(8):852–7. 10.1038/s41587-019-0209-9 . 31341288 PMC7015180

[20] Callahan BJ, McMurdie PJ, Rosen MJ, Han AW, Johnson AJA, Holmes SP. DADA2: High-resolution sample inference from Illumina amplicon data. Nat Methods. 2016;13(7):581–+. 10.1038/nmeth.3869 WOS:000383794500017. 27214047 PMC4927377

[21] Katoh K, Misawa K, Kuma K, Miyata T. MAFFT: a novel method for rapid multiple sequence alignment based on fast Fourier transform. Nucleic Acids Res. 2002;30(14):3059–66. 10.1093/nar/gkf436 WOS:000177154300016. 12136088 PMC135756

[22] Price MN, Dehal PS, Arkin AP. FastTree 2-Approximately Maximum-Likelihood Trees for Large Alignments. Plos One. 2010;5(3). 10.1371/journal.pone.0009490 e949010.1371/journal.pone.0009490. WOS:000275328800002. 20224823 PMC2835736

[23] Bokulich NA, Kaehler BD, Rideout JR, Dillon M, Bolyen E, Knight R, et al Optimizing taxonomic classification of marker-gene amplicon sequences with QIIME 2's q2-feature-classifier plugin. Microbiome. 2018;6(1):90 10.1186/s40168-018-0470-z 29773078 PMC5956843

[24] DeSantis TZ, Hugenholtz P, Larsen N, Rojas M, Brodie EL, Keller K, et al Greengenes, a chimera-checked 16S rRNA gene database and workbench compatible with ARB. Appl Environ Microbiol. 2006;72(7):5069–72. 10.1128/AEM.03006-05 16820507 PMC1489311

[25] Douglas GM, Maffei VJ, Zaneveld JR, Yurgel SN, Brown JR, Taylor CM, et al PICRUSt2 for prediction of metagenome functions. Nat Biotechnol. 2020 10.1038/s41587-020-0548-6 . 32483366 PMC7365738

[26] SAS Institute. SAS User’s Guide. Statistics, Version 9.2. Cary, NC: SAS Inst Inc; 2009.

[27] Zakrzewski M, Proietti C, Ellis JJ, Hasan S, Brion MJ, Berger B, et al Calypso: a user-friendly web-server for mining and visualizing microbiome-environment interactions. Bioinformatics. 2017;33(5):782–3. 10.1093/bioinformatics/btw725 WOS:000397265300030. 28025202 PMC5408814

[28] Hughes JB, Hellmann JJ, Ricketts TH, Bohannan BJ. Counting the uncountable: statistical approaches to estimating microbial diversity. Appl Environ Microbiol. 2001;67(10):4399–406. 10.1128/aem.67.10.4399-4406.2001 11571135 PMC93182

[29] Kim BR, Shin J, Guevarra R, Lee JH, Kim DW, Seol KH, et al Deciphering diversity indices for a better understanding of microbial communities. J Microbiol Biotechnol. 2017;27(12):2089–93. 10.4014/jmb.1709.09027 . 29032640

[30] Lozupone C, Lladser ME, Knights D, Stombaugh J, Knight R. UniFrac: an effective distance metric for microbial community comparison. ISME J. 2011;5(2):169–72. 10.1038/ismej.2010.133 20827291 PMC3105689

[31] Goodrich JK, Di Rienzi SC, Poole AC, Koren O, Walters WA, Caporaso JG, et al Conducting a microbiome study. Cell. 2014;158(2):250–62. 10.1016/j.cell.2014.06.037 25036628 PMC5074386

[32] Fukuyama J. Emphasis on the deep or shallow parts of the tree provides a new characterization of phylogenetic distances. Genome Biol. 2019;20(1):131 10.1186/s13059-019-1735-y 31253178 PMC6599322

[33] Hall JA, Van Saun RJ, Nichols T, Mosher W, Pirelli G. Comparison of selenium status in sheep after short-term exposure to high-selenium-fertilized forage or mineral supplement. Small Ruminant Res. 2009;82(1):40–5. 10.1016/j.smallrumres.2009.01.010 ISI:000264942500007.

[34] Wallace LG, Bobe G, Vorachek WR, Dolan BP, Estill CT, Pirelli GJ, et al Effects of feeding pregnant beef cows selenium-enriched alfalfa hay on selenium status and antibody titers in their newborn claves. J Anim Sci. 2017;95:2408–20. 10.2527/jas.2017.1377 28727057 PMC7114777

[35] Hall JA, Isaiah A, Estill CT, Pirelli GJ, Suchodolski JS. Weaned beef calves fed selenium-biofortified alfalfa hay have an enriched nasal microbiota compared with healthy controls. PLoS One. 2017;12(6):e0179215 Epub 2017/06/09. 10.1371/journal.pone.0179215 28594919 PMC5464631

[36] NRC. Nutrient Requirements of Beef Cattle: Seventh Revised Edition: Update 2000. Washington DC: National Academy Press, 2000.

[37] Whanger PD. Selenocompounds in plants and animals and their biological significance. J Am Coll Nutr. 2002;21(3):223–32. Epub 2002/06/21. 10.1080/07315724.2002.10719214 . 12074249

[38] Swecker WS Jr., Hunter KH, Shanklin RK, Scaglia G, Fiske DA, Fontenot JP. Parenteral selenium and vitamin E supplementation of weaned beef calves. J Vet Intern Med. 2008;22(2):443–9. 10.1111/j.1939-1676.2008.0054.x . 18346143

[39] Mehdi Y, Dufrasne I. Selenium in Cattle: A Review. Molecules. 2016;21(4):545 10.3390/molecules21040545 27120589 PMC6274551

[40] Hooper KJ, Bobe G, Vorachek WR, Bishop-Stewart JK, Mosher WD, Pirelli GJ, et al Effect of selenium yeast supplementation on naturally acquired parasitic infection in ewes. Biol Trace Elem Res. 2014;161(3):308–17. Epub 2014/09/27. 10.1007/s12011-014-0134-1 . 25256922

[41] Colditz IG, Watson DL, Gray GD, Eady SJ. Some relationships between age, immune responsiveness and resistance to parasites in ruminants. Int J Parasitol. 1996;26(8–9):869–77. 10.1016/s0020-7519(96)80058-0 . 8923136

[42] Proctor DM, Relman DA. The landscape ecology and microbiota of the human nose, mouth, and throat. Cell Host Microbe. 2017;21(4):421–32. 10.1016/j.chom.2017.03.011 WOS:000398896100004. 28407480 PMC5538306

[43] Stroebel C, Alexander T, Workentine ML, Timsit E. Effects of transportation to and co-mingling at an auction market on nasopharyngeal and tracheal bacterial communities of recently weaned beef cattle. Vet Microbiol. 2018;223:126–33. 10.1016/j.vetmic.2018.08.007 WOS:000445310400019. 30173738

[44] Holman DB, Timsit E, Booker CW, Alexander TW. Injectable antimicrobials in commercial feedlot cattle and their effect on the nasopharyngeal microbiota and antimicrobial resistance. Vet Microbiol. 2018;214:140–7. 10.1016/j.vetmic.2017.12.015 . 29408026

[45] McMullen C, Orsel K, Alexander TW, van der Meer F, Plastow G, Timsit E. Evolution of the nasopharyngeal bacterial microbiota of beef calves from spring processing to 40 days after feedlot arrival. Vet Microbiol. 2018;225:139–48. 10.1016/j.vetmic.2018.09.019 . 30322526

[46] Timsit E, Workentine M, van der Meer F, Alexander T. Distinct bacterial metacommunities inhabit the upper and lower respiratory tracts of healthy feedlot cattle and those diagnosed with bronchopneumonia. Vet Microbiol. 2018;221:105–13. 10.1016/j.vetmic.2018.06.007 . 29981695

[47] McMullen C, Orsel K, Alexander TW, van der Meer F, Plastow G, Timsit E. Comparison of the nasopharyngeal bacterial microbiota of beef calves raised without the use of antimicrobials between healthy calves and those diagnosed with bovine respiratory disease. Vet Microbiol. 2019;231:56–62. 10.1016/j.vetmic.2019.02.030 . 30955824

[48] Hall JA, Sendek RL, Chinn RM, Bailey DP, Thonstad KN, Wang Y, et al Higher whole-blood selenium is associated with improved immune responses in footrot-affected sheep. Vet Res. 2011;42(1):99 Epub 2011/09/08. 10.1186/1297-9716-42-99 [pii]. 21896161 PMC3179948

[49] Hugejiletu H, Bobe G, Vorachek WR, Gorman ME, Mosher WD, Pirelli GJ, et al Selenium supplementation alters gene expression profiles associated with innate immunity in whole-blood neutrophils of sheep. Biol Trace Elem Res. 2013;154(1):28–44. Epub 2013/06/12. 10.1007/s12011-013-9716-6 . 23754590

[50] Bankvall M, Jontell M, Wold A, Ostman S. Tissue-specific differences in immune cell subsets located in the naso-oropharyngeal-associated lymphoid tissues. Scand J Immunol. 2018;87(1):15–27. 10.1111/sji.12625 . 29077981

[51] Ganger S, Schindowski K. Tailoring formulations for intranasal nose-to-brain delivery: a review on architecture, physico-chemical characteristics and mucociliary clearance of the nasal olfactory mucosa. Pharmaceutics. 2018;10(3). 10.3390/pharmaceutics10030116 30081536 PMC6161189

[52] Willing BP, Gill N, Finlay BB. The role of the immune system in regulating the microbiota. Gut Microbes. 2010;1(4):213–23. 10.4161/gmic.1.4.12520 21327028 PMC3023603

[53] Taherali F, Varum F, Basit AW. A slippery slope: On the origin, role and physiology of mucus. Adv Drug Deliv Rev. 2018;124:16–33. 10.1016/j.addr.2017.10.014 . 29108861

[54] Zeineldin M, Lowe J, de Godoy M, Maradiaga N, Ramirez C, Ghanem M, et al Disparity in the nasopharyngeal microbiota between healthy cattle on feed, at entry processing and with respiratory disease. Vet Microbiol. 2017;208:30–7. 10.1016/j.vetmic.2017.07.006 . 28888646

[55] Parker AM, Sheehy PA, Hazelton MS, Bosward KL, House JK. A review of mycoplasma diagnostics in cattle. J Vet Intern Med. 2018;32(3):1241–52. 10.1111/jvim.15135 29671903 PMC5980305

[56] Christodoulides A, Gupta N, Yacoubian V, Maithel N, Parker J, Kelesidis T. The role of lipoproteins in mycoplasma-mediated immunomodulation. Front Microbiol. 2018;9:1682 10.3389/fmicb.2018.01682 30108558 PMC6080569

[57] Kuhn MJ, Hopkins SM. A clinical review of bovine ureaplasmosis. Iowa State University Veterinarian. 1983;45(1):20–4.

[58] Silva JK, Marques LM, Timenetsky J, de Farias ST. Ureaplasma diversum protein interaction networks: evidence of horizontal gene transfer and evolution of reduced genomes among Mollicutes. Can J Microbiol. 2019;65(8):596–612. 10.1139/cjm-2018-0688 . 31018106

[59] Syrala P, Anttila M, Dillard K, Fossi M, Collin K, Nylund M, et al Causes of bovine abortion, stillbirth and neonatal death in Finland 1999–2006. Acta Veterinaria Scandinavica. 2007;49 Artn S3 10.1186/1751-0147-49-S1-S3 WOS:000254063200004.

[60] Nascimento-Rocha JM, Oliveira BDF, Arnhold E, Porto RNG, Lima SF, Gambarini ML. Assessment of cow and farm level risk factors associated with Ureaplasma diversum in pasture-based dairy systems—A field study. An Acad Bras Cienc. 2017;89(3):1779–83. 10.1590/0001-3765201720160813 . 28876387

[61] McMullen C, Alexander TW, Leguillette R, Workentine M, Timsit E. Topography of the respiratory tract bacterial microbiota in cattle. Microbiome. 2020;8(1):91 10.1186/s40168-020-00869-y 32522285 PMC7288481

[62] ter Laak EA, Noordergraaf JH, Dieltjes RP. Prevalence of mycoplasmas in the respiratory tracts of pneumonic calves. Zentralbl Veterinarmed B. 1992;39(8):553–62. 10.1111/j.1439-0450.1992.tb01205.x . 1462720

[63] Tegtmeier C, Uttenthal A, Friis NF, Jensen NE, Jensen HE. Pathological and microbiological studies on pneumonic lungs from Danish calves. Zentralbl Veterinarmed B. 1999;46(10):693–700. 10.1046/j.1439-0450.1999.00301.x 10676147 PMC7183811

[64] Marques LM, Rezende IS, Barbosa MS, Guimaraes AM, Martins HB, Campos GB, et al Ureaplasma diversum genome provides new insights about the interaction of the surface molecules of this bacterium with the host. PLoS One. 2016;11(9):e0161926 10.1371/journal.pone.0161926 27603136 PMC5015763

[65] Andrade Y, Santos-Junior MN, Rezende IS, Barbosa MS, Amorim AT, Silva IBS, et al Multilocus sequence typing characterizes diversity of Ureaplasma diversum strains, and intra-species variability induces different immune response profiles. BMC Vet Res. 2020;16(1):163 10.1186/s12917-020-02380-w 32456681 PMC7249313

[66] Manso-Silvan L, Dupuy V, Lysnyansky I, Ozdemir U, Thiaucourt F. Phylogeny and molecular typing of Mycoplasma agalactiae and Mycoplasma bovis by multilocus sequencing. Vet Microbiol. 2012;161(1–2):104–12. 10.1016/j.vetmic.2012.07.015 . 22841405

[67] Bottinelli M, Passamonti F, Rampacci E, Stefanetti V, Pochiero L, Coletti M, et al DNA microarray assay and real-time PCR as useful tools for studying the respiratory tract Mycoplasma populations in young dairy calves. J Med Microbiol. 2017;66(9):1342–9. 10.1099/jmm.0.000571 . 28868997

[68] McDaneld TG, Kuehn LA, Keele JW. Evaluating the microbiome of two sampling locations in the nasal cavity of cattle with bovine respiratory disease complex (BRDC). J Anim Sci. 2018 10.1093/jas/sky032 29659872 PMC6140963

[69] McDaneld TG, Kuehn LA, Keele JW. Microbiome of the upper nasal cavity of beef calves prior to weaning. J Anim Sci. 2019;97(6):2368–75. 10.1093/jas/skz119 31144716 PMC6541832

[70] Timsit E, Holman DB, Hallewell J, Alexander TW. The nasopharyngeal microbiota in feedlot cattle and its role in respiratory health. Animal Frontiers. 2016;6(2):44–50. 10.2527/af.2016-0022

